# Improvement of the froth flotation of LiAlO_2_ and melilite solid solution via pre-functionalization

**DOI:** 10.1038/s41598-021-00008-z

**Published:** 2021-10-14

**Authors:** Hao Qiu, Jule Kersebaum, Annett Wollmann, Niklas Feuge, Andrea Haas, Daniel Goldmann, René Wilhelm

**Affiliations:** 1 Institute of Mineral and Waste Processing, Waste Disposal and Geotechnics, Clausthal University of Technology, Walther-Nernst-Str. 9, 38678 Clausthal-Zellerfeld, Germany; 2 Institute of Particle Technology, Clausthal University of Technology, Leibnizstr. 19, 38678 Clausthal-Zellerfeld, Germany; 3 Institute of Organic Chemistry, Clausthal University of Technology, Leibnizstr. 6, 38678 Clausthal-Zellerfeld, Germany

**Keywords:** Surface assembly, Chemical engineering, Chemical engineering

## Abstract

In this work froth flotation studies with LiAlO_2_ (lithium-containing phase) and Melilite solid solution (gangue phase) are presented. The system was optimized with standard collectors and with compounds so far not applied as collectors. Furthermore, the principle of self-assembled monolayers was introduced to a froth flotation process for the first time resulting in excellent yields and selectivities.

## Introduction

With the development and extension of lithium-ion batteries, electric vehicles, the demand for Lithium resources is significantly increasing. In the end market, the share of lithium consumption for batteries increases from 65% in 2019 to 71% in 2020^1–3^. In 2020, the worldwide consumption of lithium content was estimated to 56,000 Tons^2^. With the current growth rate, a potential shortage of raw materials could threaten supply safety. Hence, efficient recovery of lithium from spent LIBs is vital.

Pyrometallurgical processing is one of the vital routes to recycle value metals from spent LIBs with the advantage that input materials can fluctuate^4^. The Umicore Battery Recycling Process is a pyrometallurgical process developed to recover NiMH and spent lithium-ion batteries^5^. Co, Ni, and Cu can be enriched in alloys while Li is transferred into slags^5^. From the composition of a Umicore slag mainly based on the Li_2_O–MgO–Al_2_O_3_–SiO_2_–CaO slag system with high aluminum content, it is observed that Li is present in the slag in the form of the LiAlO_2_^6^. LiAlO_2_ contains about 10% lithium, while the critical lithium-bearing mineral Spodumene contains only 3.7% lithium. Besides, Spodumene needs to be converted from α-Spodumene to β-Spodumene by roasting in the subsequent hydrometallurgical processing, a process that requires substantial amounts of energy^7^. The hydrometallurgical processing of LiAlO_2_ enriched silicate slag has also shown that Li recovery can reach 80–95%^5^. Therefore, in comparison, LiAlO_2_ is more economically advantageous.

Among several known lithium-containing slags based on the Li_2_O–MgO–Al_2_O_3_–SiO_2_–CaO slag system, LiAlO_2_ is present in the slags as a lithium-bearing phase^4,6,8^. Melilite solid solution (Melilite s.s.) also appears in the slags as a gangue mineral^4,6^. As a typical slag mineral, its two common end members, gehlenite (Ca_2_Al(AlSi)O_7_) and åkermanite (Ca_2_MgSi_2_O_7_), which belongs to the melilite group, often forms a continuous solid solution^9–13^. Since LiAlO_2_ is a critical lithium-bearing phase in lithium-bearing slag, a systematic study of the floatability of LiAlO_2_ and its common gangue minerals in lithium-bearing slags is crucial for the improvement of the pyrometallurgical recovery path for lithium-ion batteries^8^. Previous research has shown that LiAlO_2_ can be floated by certain fatty acids (Clariant, Flotinor FS-2 and FS-100) and a phosphoric acid ester (Clariant, Flotinor SM-15)^14^. However, in the real slag, both the lithium yield (approx.60%) and the enrichment factors (approx. 1.5) were insufficient^14^.

Froth flotation is based on the selective interaction of collectors with surfaces of different minerals. This adsorption of the collectors can occur via physisorption or chemisorption^15^. It is possible to state that during froth flotation in situ functionalization via physisorption and chemisorption occurs, which is selectively increasing the hydrophobicity of different minerals surfaces. Besides van der Waals interactions and hydrogen bonds^16^, electrostatic forces are the predominant cause of physical adsorption^17^ of collectors in froth flotation^18^. The formation of a dative bond between a collector and a metal ion is the reason for chemisorption^19^, assuming that the stability constants of the “complexes” on the surface in aqueous solution are very far on the product side or the complexed area has become inert due to hydrophobic repulsion of water. In addition, during the froth flotation in water an electrical charge equilibrium is formed^20,21^ and different oxides and silicate minerals are partially dissolved, resulting in a change of the pH and a “fresh surface” where metal ions may be coordinated by water molecules or by collector molecules via formation of dative bonds^21^. Next to collectors and pH values, froth flotation can also be influenced by frothers, modifiers, depressants, and flocculants^22^. Dissolved metallic ions from metal oxides can also influence the outcome of a froth flotation. They can adsorb on the surface of silicates binding to their hydroxy groups and influencing their flotation behavior^20,23^. Although the in situ functionalization in water is highly complex, with various parameters to be considered, froth flotation is an established method for enrichment and separation of different minerals since the end of the nineteenth century^22^.

However, there is also another way to increase the hydrophobicity of mineral surfaces. Under ambient conditions, there is always a layer of adsorbed water present on metal oxides, which leads also on metal ion terminated surfaces to the formation of hydroxyl groups^24–26^. Hence, besides a few defect sides on the surfaces, the predominant terminal functional groups on minerals are hydroxyl moeties^27^. This is utilized in the research field of Self-Assembled Monolayers (SAM) where most commonly condensation reactions in organic solvents between linker groups (also called anchoring groups) and the hydroxyl groups of oxide surfaces create covalent bonds^28^. Functionalized materials with a high surface area have a high potential in fields such as drug delivery^29–31^, separation^32^, sensing^33^, nanotechnology^34^, or heterogeneous catalysis^3,35,36^. Hence, they have been functionalized for these applications with different molecules. Taking the latter into account, we present here an alternative approach towards froth flotation and compare the behavior of different collectors in an in situ standard flotation to a pre-functionalization approach.

## Experimental details

### Materials

Sodium oleate (Riedel-de-Haën), naphthenic acid (Fluka), pine oil (American Cyanamid), bis(2-ethylhexyl) phosphate, dodecyl phosphonic acid (TCI), dibutyl phosphate (Sigma-Aldrich), thenoyltrifluoroacetone (TCI), and trioctyl phosphine oxide (Sigma-Aldrich) were purchased. Naphthenic acid is saponified by adding NaOH to obtain sodium naphthenate. Decyl dihydrogen phosphate^37^ was prepared according to literate procedures. LiAlO_2_ (Sigma-Aldrich) and Melilite s.s. were separately used for flotation experiments. The melilite s.s. ore was obtained from Vata de Sus, located in Hunedoara, Romania. It was firstly crushed with a hammer, then ground in a rod mill, and screened in a stainless-steel screen. The under the size of 63 μm sieve product was selected for flotation tests. LiAlO_2_ was also sieved, and the under the size of 63 μm sieve product was selected for flotation tests. BET measurements revealed that the sieved LiAlO_2_ had a BET surface of < 1 m^2^/g and Melilite s.s. of 3.5 m^2^/g. X-ray powder diffraction measurement and chemical analysis were performed on the mineral samples. The chemical composition was listed in Table 1. According to XRD analysis, the main phase of the ore is Melilite s.s., and also, there are some impurities such as Merwinite, Wollastonite and Calcite.

**Table 1:** Chemical composition of Melilite s.s.

	Sum (%)
Na_2_O	0.2
MgO	4.1
Al_2_O_3_	15.99
SiO_2_	27.49
P_2_O_5_	0.02
Cl	0.08
K_2_O	0.02
CaO	31.88
TiO_2_	0.409
MnO	0.04
Fe_2_O_3_	3.06

### Analysis

ATR-IR-Spectra were recorded on an Alpha-T IR (Bruker) with a platinum ATR-unit and diamond crystal. Vibrations are given in cm^−1^.

### Flotation

**Figure 1:**
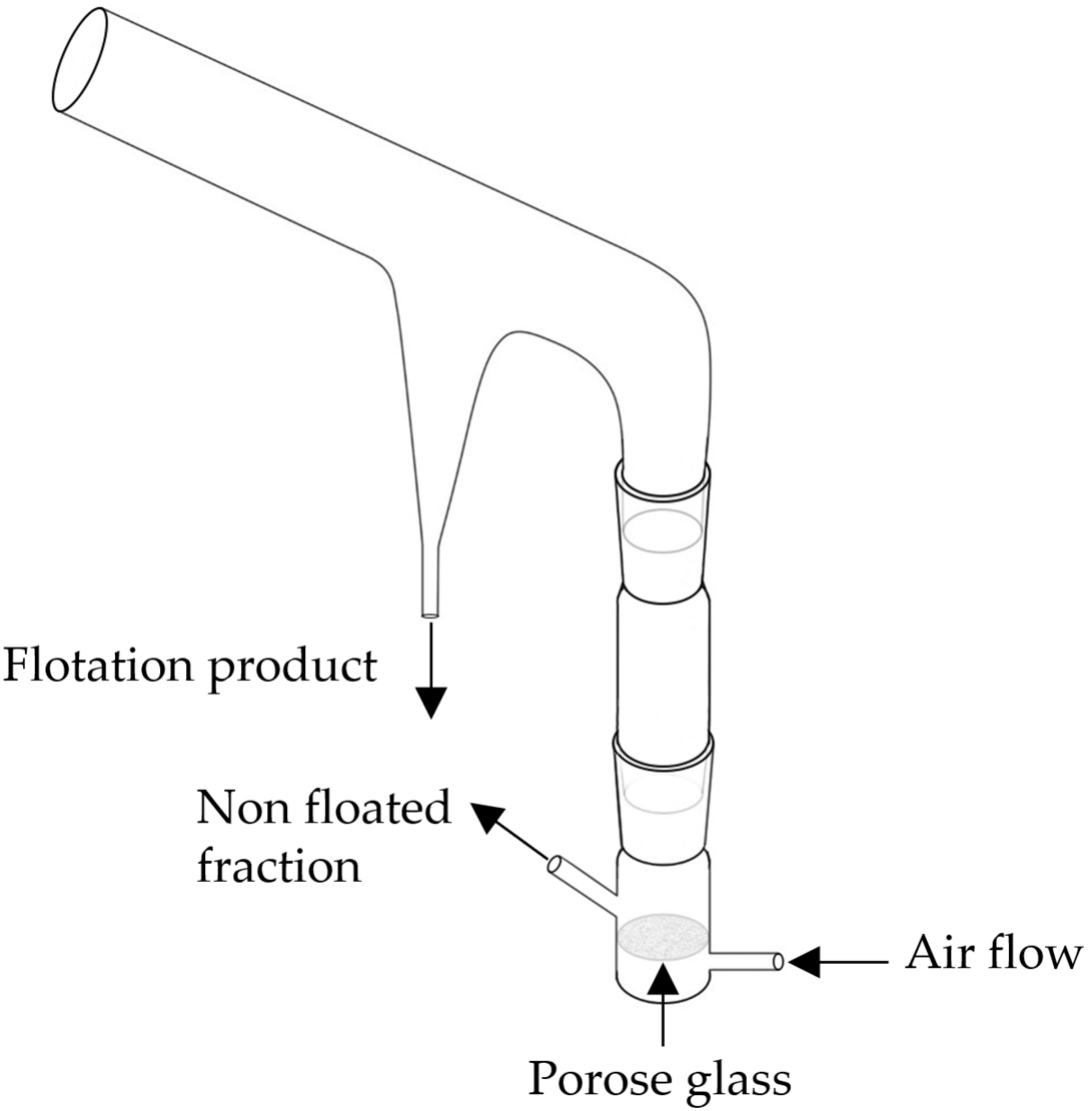
Sketch of the modified Hallimond tube.

The flotation test was performed using a modified Hallimond tube (Fig. 1), consisting of three parts: the upper part, the extended part, and the bottom part, produced by HI-ALOQUIMICA, Brazil. All three parts can be freely disassembled for easy cleaning. A piece of porous glass (medium-pore fritted glass) is installed in the bottom part, through which the airflow can enter the tube. A small magnetic stir bar can be placed at the bottom of the Hallimond tube to stir the slurry. The sample amount for each test was 2 g, and the test was carried out at ambient temperature with pine oil (150 g/t) as frother. Firstly, the slurry was mixed and stirred in a beaker for 1 min before the dosing, followed by 1 min after dosing for conditioning. Then the slurry was transferred to Hallimond tube for flotation. The flotation time lasted for 3 min. The airflow rate of each test was controlled to be 1.89 L/h, and the rotation speed was controlled to be 500 rpm. The pretreated samples were not added with collectors. Since LiAlO_2_ is hydrolyzed in water, its natural pH is about 11. In the meantime, LiAlO_2_ continuously reacts with sulfuric acid to generate lithium sulfate, making it difficult to adjust the pH to a stable state. Therefore, in this study, the pH value was stabilized using the Britton-Robinson buffer, which consisted of 0.04 M boric acid, 0.04 M phosphoric acid, and 0.04 M acetic acid, and the required pH was achieved by the addition of 0.2 M NaOH.

### General functionalization

LiAlO_2_ or Melilite s.s., 10 w% of organic compound and toluene were refluxed under N_2_ in a Dean–Stark apparatus for 16 h or stirred at room temperature for 16 h. Thereafter, the toluene was filtered from the solid. The remaining solid was washed three times with toluene and dried under high vacuum. The combined toluene solutions were evaporated and non-reacted organic compounds were recovered.

## Results and discussion

**Figure 2:**
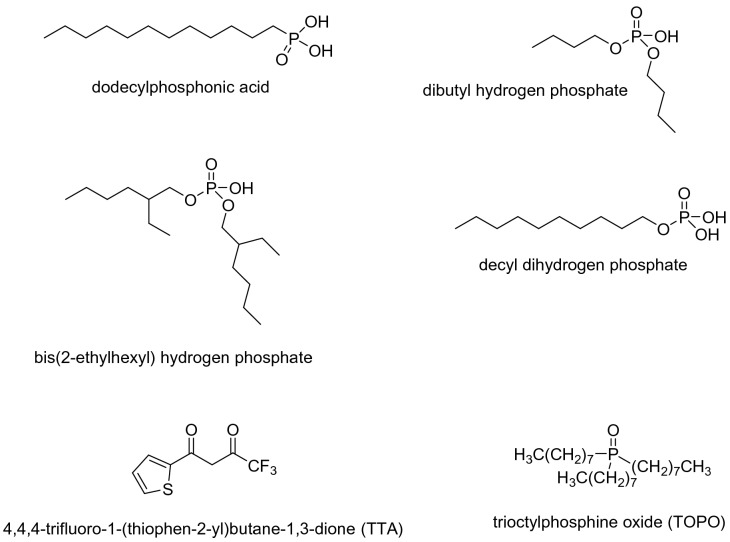
Used collector compounds next to sodium oleate.

The investigation started with the functionalization of LiAlO_2_ as the lithium containing phase and Melilite s.s. as the gangue phase. Each functionalized material was applied in froth flotation and, where possible, compared with the in situ process. Next to sodium oleate further known collectors and compounds, which have not been explored as collectors so far, were used in the froth flotation and are depicted in Fig. 2.

Alkyl phosphonic acids like dodecyl phosphonic acid have been applied for surface functionalizations as self-assemble layers^28^ in order to obtain organic electronics^38^, material for liquid chromatography^39^, to immobilized catalysts^40^, or to protect different surfaces of metal oxides^41–44^. A collection of possible types of attachment to oxide surface is shown in Scheme 3^34^. In one case where dodecyl phosphonic acid was applied for the passivation of aluminum and its alloys the quality of the linker group was tested by pH-dependent floating tests^45^. Phosphonic acids are moisture-stable and easy to handle. They have nowadays become standard linkers for various oxidic support materials^46,47^. On the other hand, alkyl phosphonic acids have not been used as collectors in froth flotation. Collectors based on esters of phosphoric acids are well established^48–51^.

For pre-functionalized samples, no collector was added in the flotation studies. For comparison a standard froth flotation was optimized for LiAlO_2_ and Melilite s.s. using sodium oleate (300 g/t) as the best collector resulting in 69% for LiAlO_2_ and 34% for Melilite s.s. compared to a flotation without a collector, which obtained LiAlO_2_ in 18% and Melilite s.s. in 16%. Best results achieved under natural pH (For further results in different collector dosages and pHs see Supplementary Information page S2). LiAlO_2_ and Melilite s.s. were functionalized with 10 w% dodecyl phosphonic acid and n-dibutyl phosphate once in refluxing toluene and once just by stirring at room temperature^40^. After filtration, the material was washed with toluene and dried. The combined toluene filtrates were combined and evaporated to dryness to recover the phosphonic acid or phosphate. In all cases both minerals became very hydrophobic to such an extent that froth flotation experiments were not possible. Nevertheless, the experiments showed that also at room temperature, a high level of functionalization is feasible.

**Figure 3:**
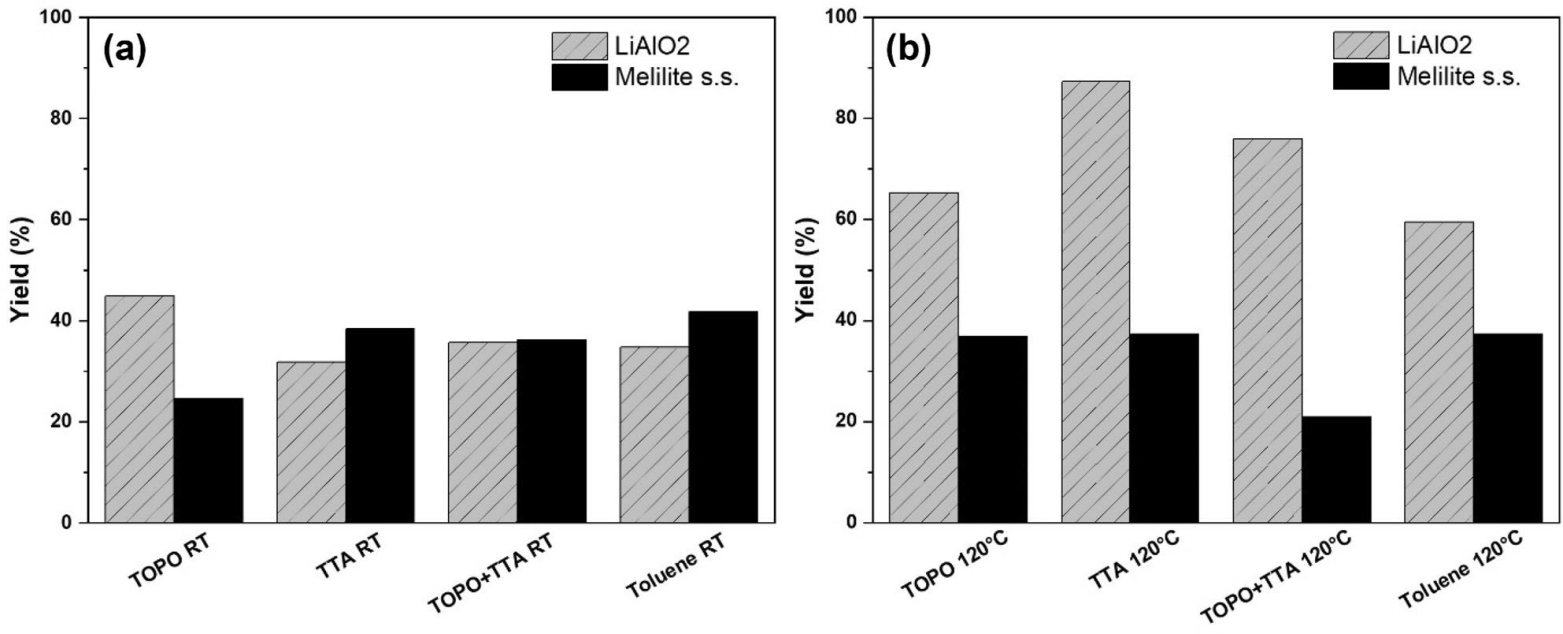
Flotation yield of TTA and TOPO functionalized LiAlO_2_ (**a**) and Melilite (**b**).

Next, we chose thenoyltrifluoroacetone (TTA) and trioctyl phosphine oxide (TOPO) for the functionalization. TTA is very well known as a complexing agent for extractions^52^ including lithium ions^53,54^ and as a ligand in various metal complexes^55,56^. It was also used in the surface modification of europium salts^57^, but not as a collector in froth flotations. TOPO was applied in the extraction of lithium ions in combination of TTA^54^ and other ligands^58^. In addition, it was utilized to functionalize, synthesize, and stabilize perovskite nanomaterial and influencing their band gaps^59–61^. The results of the flotation experiments are shown in Fig. 3. The flotation yield of LiAlO_2_, functionalized with TTA at 120 °C, increased to 87% and to 37% for Melilite s.s. The yield of LiAlO_2_ can be increased to 76% and that of Melilite s.s. to 21% if the functionalization is carried out with a 1:1 mixture of TOPO and TTA. The flotation yields and selectivities in most cases decreased significantly when a functionalization was attempted at room temperature.

It is not possible to compare TTA, TOPO or dodecyl phosphonic acid with a standard froth flotation system and apply them as collectors since they are solids and almost insoluble in water. Hence, sodium oleate was applied in the functionalization experiments in refluxing toluene which provided a yield in the flotation for LiAlO_2_ of 62%, and for Melilite s.s. of 21% (see Supplementary Information page S18, Figure S25). These results are slightly off target compared to the standard process. It is reasonable to assume that this is due to desorption of the carboxylate in water. The functionalization with the carboxylate salt primarily proceeds via formation of hydrogen bonds. To form ester bonds the free carboxylic acid is needed^62^.

**Figure 4:**
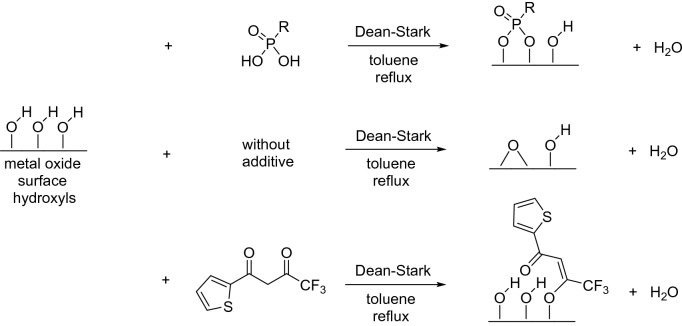
Functionalization with LiAlO_2_ and Melilite s.s.

**Figure 5:**
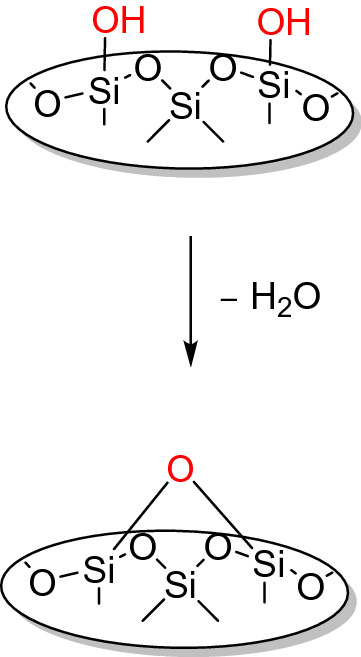
Self-dehydroxylation on silica under water removing.

In Fig. 4, some suggestions are given for possible functionalization modes leading to surfaces with enhanced hydrophobicity, which correlates to the flotation yield. Figure 3 shows that the flotation yield of primarily LiAlO_2_ already increases by just refluxing the material in toluene in a Dean-Stark apparatus. The expected surface reaction is given in Fig. 4 and in more detail in Fig. 5.

**Figure 6:**
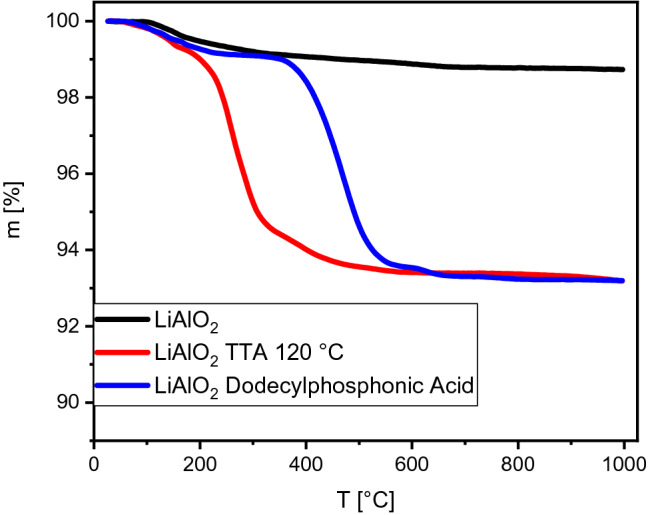
Selected TGA measurements of functionalized LiAlO_2_ conditions^63^.

It is well known for silica that a self-condensation (i.e. self-dehydroxylation) can occur under water-removing conditions and that this new surface only reacts slowly (weeks) with moisture back to its original structure^63^. This new surface, however, can participate only as a passive partner in a hydrogen bond, hence, the surface is less hydrophilic compared to a surface with hydroxyl groups. It is possible to argue that the increase of the yield of the TTA functionalized material is just adding up to the yield of a dehydroxylated surface. However, from TGA measurements shown in Fig. 6, it is possible to calculate a high loading of TTA molecules on LiAlO_2_. The loading level was confirmed with elemental analysis results. TGA measurements with Melilite s.s. were not possible due to the high water content of the silicate, hence also elemental analysis was carried out for functionalized LiAlO_2_ and Melilite s.s. In the case of LiAlO_2_, elemental analysis results were consistent with TGA studies, and in all cases, LiAlO_2_ showed a much higher level of functionalization than Melilite s.s. (For further details on TGA and Elemental analysis results, see Supplementary Information, page S7). The proposed structure of TTA on LiAlO_2_ in Fig. 4 would also occur when TTA is reacting with the quasi-condensed epoxide-type surface. The TGA measurements of dodecyl phosphonic acid are in very good agreement with the literature^64^, where first the bond between the phosphorous atom and the alkyl chain is cleaved. Also, the presence of one covalent bond for TTA is supported by the TGA results.

**Figure 7:**
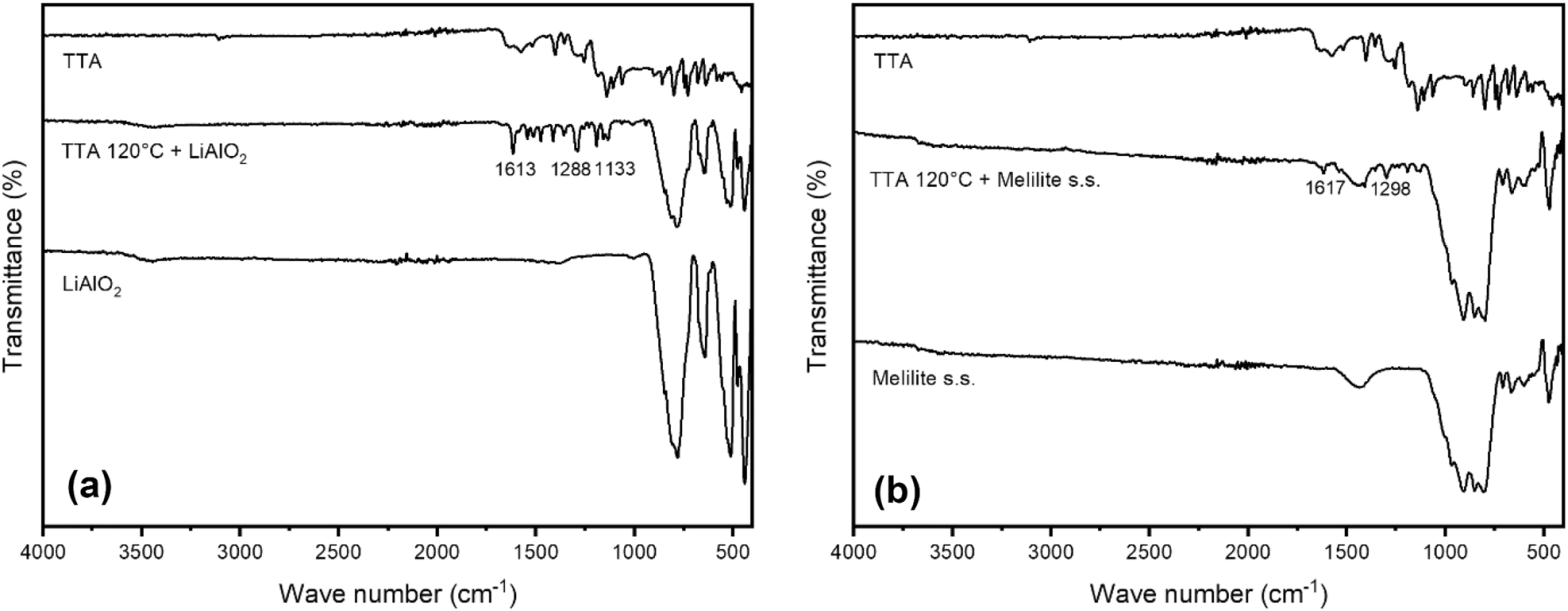
FT-IR measurement of TTA functionalized LiAlO_2_ (**a**) and Melilite s.s. (**b**).

FT-IR measurements were also conducted to get further inside on the level of functionalization of each material. As can be seen in Fig. 7, the signals of TTA are found in the functionalized material. The signals are significantly stronger with LiAlO_2_ as with Melilite s.s. Taking into account the unfavorable ratio of the surface to the rest of the material the FT-IR spectra are not intensive enough to prove the exact mode of functionalization as suggested in Fig. 4. However, this is in the field of SAM for bulk material a common phenomenon^40^. Yet, the presence of sharp peaks at the functionalized material compared to TTA suggest that a strong ordered functionalization took place. (For further FT-IR spectra see Supplementary Information, page S9).

Considering that phosphorous esters and phosphonic acids resulted into functionalized material too hydrophobic for froth flotation studies, decyl dihydrogen phosphate and bis (2-ethylhexyl) phosphate were investigated under standard froth flotation conditions using the Hallimond tube. Decyl dihydrogen phosphate (Fig. 2) can be simply prepared from decanol and phosphorus oxylchloride^37^. It is used as extractant^65^, and there are just limited reports where it was applied in the flotation cassiterite^48^ and manganese based ores^49^. Bis(2-ethylhexyl) phosphate (Fig. 2) has a large van der Waals Radius around the phosphate moiety due to its two-branched alkyl moieties. This and the fact that only one acidic hydroxy group at the phosphate moiety remains could influence the interaction of the molecule with different ores. Bis(2-ethylhexyl) phosphate was extensively applied as an extractant for different metal ions^66,67^, including also lithium when applied in combination with tributyl phosphate^68^. In addition, some calcium minerals and sphalerite have been investigated with bis(2-ethylhexyl) phosphate in flotation experiments^50,51^. However, with lithium-containing minerals so far flotation experiments with these collectors, have not been reported so far.

**Figure 8:**
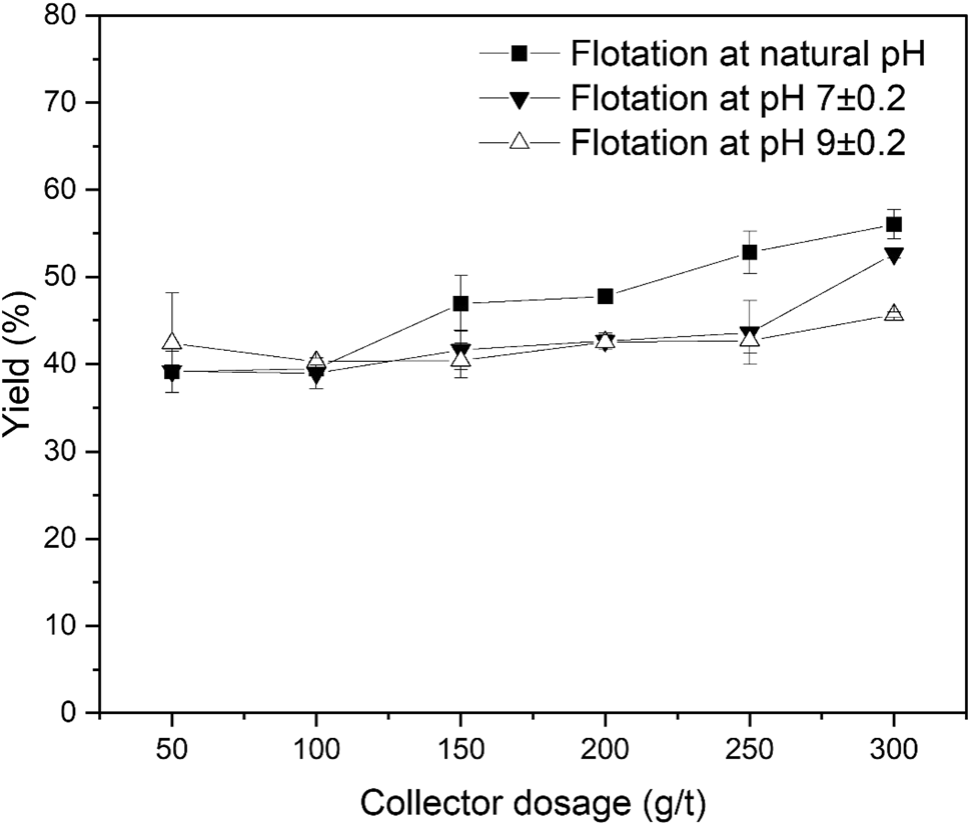
Effect of Collector Dosage on Flotation of LiAlO_2_ using decyl dihydrogen phosphate.

**Figure 9:**
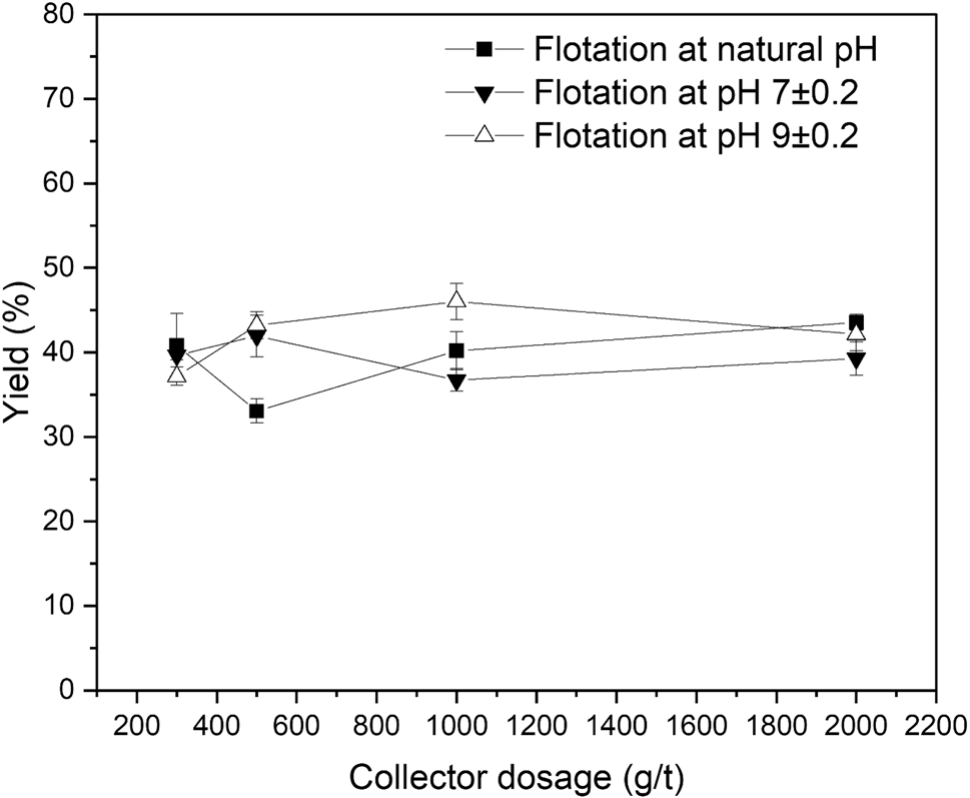
Effect of Collector Dosage on Flotation of Melilite s.s. using decyl dihydrogen phosphate.

The effect of collector dosage on the flotation of LiAlO_2_ and Melilite s.s. with decyl dihydrogen phosphate is shown in Figs. 8 and 9 at ambient temperature and at natural pH, pH 7, and pH 9. The yield increased slowly with the rise of the collector dosage and reached its maximum (56.03%) at natural pH. Its highest yield is lower than that of the sodium oleate system. This can be explained by considering that decyl dihydrogen phosphate is a sterical non-hindered phosphoric mono-ester and hydrolysis can occur too fast, resulting in the formation of an alcohol and phosphoric acid.

Figure 9 presents the effects of collector dosage on Melilite s.s. yield in the Hallimond tube flotation with decyl dihydrogen phosphate at ambient temperature and at natural pH, pH 7 and pH 9. Combining Figs. 8 and 9, it is possible to note that for the flotation of LiAlO_2_, decyl dihydrogen phosphate has a low selectivity. The yield of Melilite s.s. with a dosage of 300 g/t is about 10% lower than that of LiAlO_2,_ which can be explained again by the fast hydrolysis of the collector.

**Figure 10:**
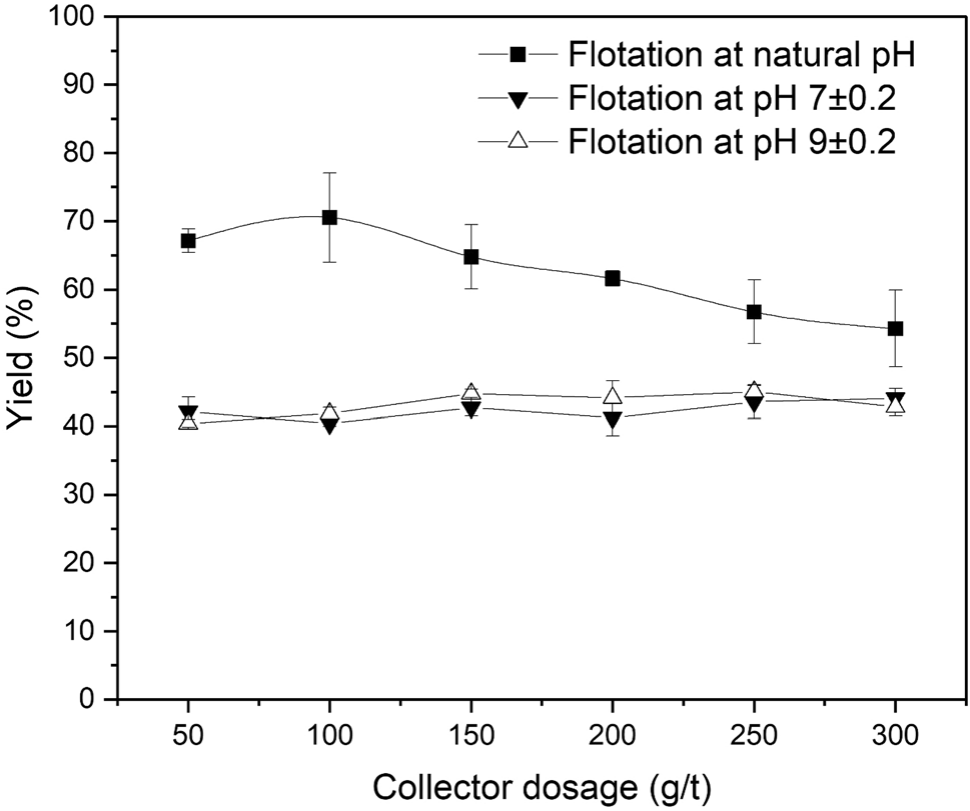
Effect of Collector Dosage on Flotation of LiAlO_2_ using bis(2-ethylhexyl) phosphate at natural pH, pH 7 and pH 9.

Thereafter the more hindered bis(2-ethylhexyl) phosphate was investigated. It was assumed that the hydrolysis rate is low enough to be applicable for the time frame during the experiment was conducted. Figure 10 presents the effects of collector dosage on LiAlO_2_ yield in the Hallimond tube flotation with bis(2-ethylhexyl) phosphate at ambient temperature and at natural pH, pH 7 and pH 9. At natural pH, the yields of LiAlO_2_ were above 60% in the first two experiments, demonstrating excellent floatability. However, in subsequent repeated experiments, one month after the first experiments, the recovery decreased to some extent. This may be related to the slow hydrolysis of the phosphate, which was stored in a ready to use aqueous solution for over a month for the experiments. In the flotation experiments at pH 7 and pH 9, the yields were very close and did not show significant changes with increasing agent dosage.

**Figure 11:**
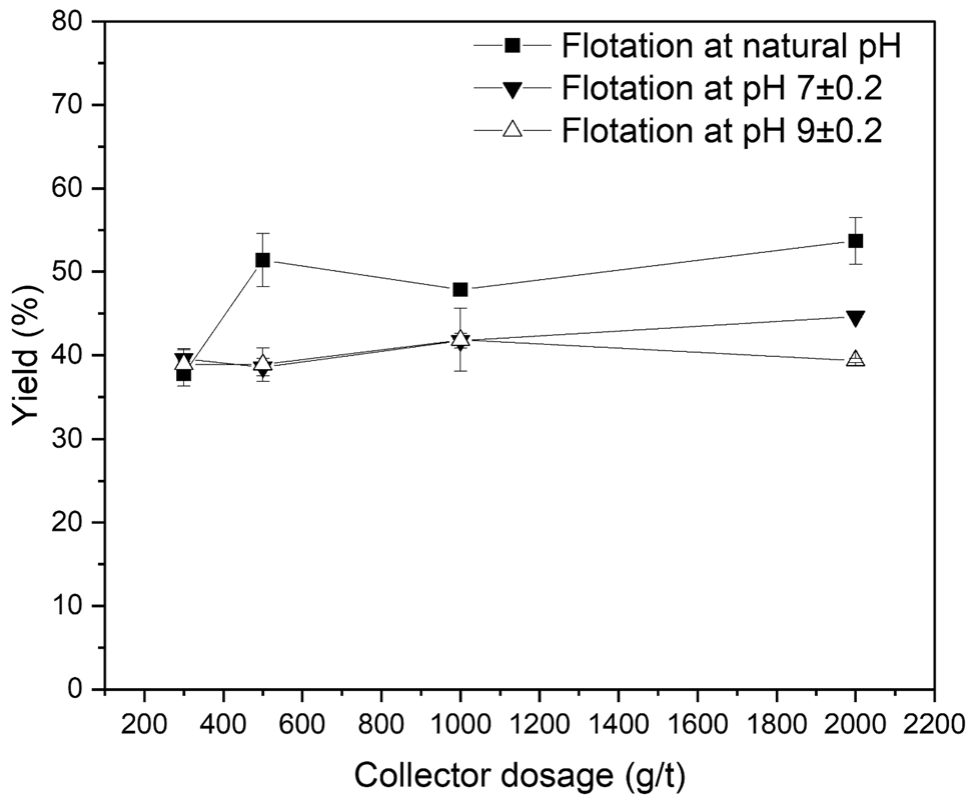
Effect of Collector Dosage on Flotation of Melilite s.s. using bis(2-ethylhexyl) phosphate.

Figure 11 presents the effects of collector dosage on Melilite s.s. yield in the Hallimond tube flotation with bis (2-ethylhexyl) phosphate at ambient temperature and at natural pH, pH 7 and pH 9. Combining Figs. 10 and 11, it is possible to see that for the flotation of LiAlO_2_, bis (2-ethylhexyl) phosphate has a certain selectivity. The collector showed an excellent recovery of LiAlO_2_ and a good selectivity.

Finally, a standard system was also scaled up in a small flotation machine. The achieved results are shown in Table 2 for LiAlO_2_ and Melilite s.s. at natural pH by using a small flotation machine.

**Table 2:** Optimized Froth Flotation with a standard collector in a small flotation machine (Denver) and pine oil (150 g/t) as frother.

Collector dosage of sodium oleate (g/t)	Yield of LiAlO_2_ (%)	Yield of Melilite s.s. (%)
150	60.32	2.88
300	91.61	7.64

**Figure 12:**
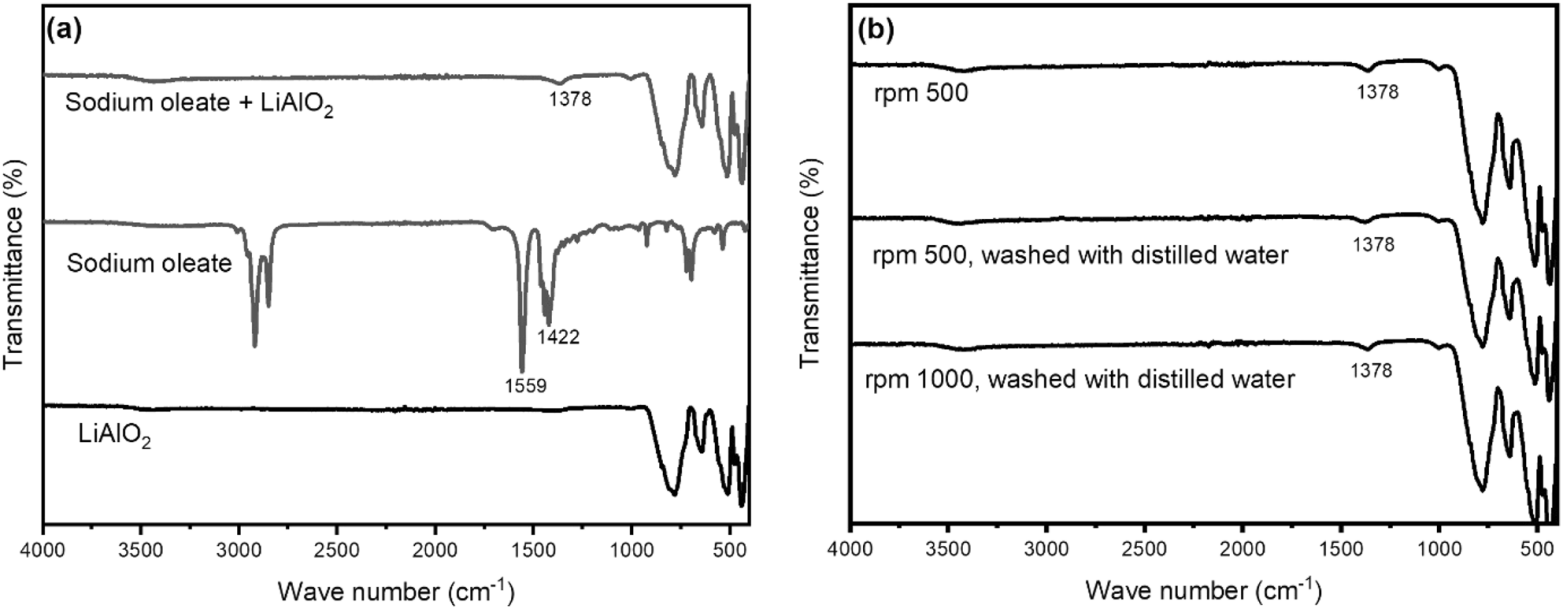
(**a**) IR spectra of LiAlO_2_, Sodium oleate and LiAlO_2_ treated in sodium oleate solution. (**b**) IR spectra of samples obtained after the desorption experiment: stirred at a rate of 500 rpm without washing; stirred at 500 rpm with washing; stirred at 1000 rpm with washing.

The IR-spectra of LiAlO_2_, sodium oleate, and LiAlO_2_ treated in 10^–5^ M sodium oleate solution are given in Fig. 12a. The bands at 1422 cm^−1^ and 1559 cm^−1^ are attributed to the symmetric and asymmetric stretching vibration of –COO– for the sodium oleate^69^. After treated with sodium oleate, the IR-spectra of LiAlO_2_ displays a new band at 1378 cm^−1^. The desorption experiment was also carried out. The new band at 1378 cm^−1^ in IR-Spectra did not disappear after strong stirring in sodium oleate solution and washing with distilled water, as shown in Fig. 12b. The appearance of this new band indicates the possibility of the formation of aluminum oleate. However, the IR measurements cannot completely display the behavior in the solution state. (For further FT-IR spectra see Supplementary Information, page S9) Contact angle measurements (Washburn) were also performed on LiAlO_2_. The contact angle of untreated LiAlO_2_ is 27.99°, while the contact angle increased to 89.49° after the addition of sodium oleate.

**Figure 13:**
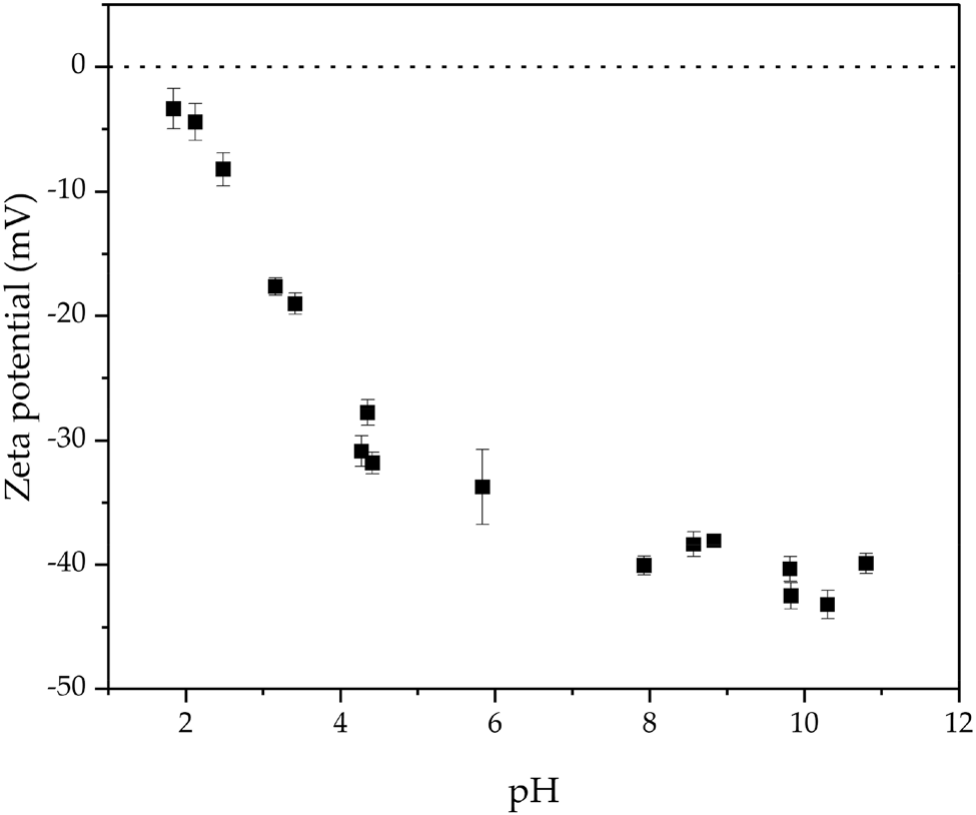
Zeta potential of Melilite s.s.

In the sodium oleate system, the overall yield of Melilite s.s. is lower than that of LiAlO_2_. As shown in Fig. 13, the Zeta potential of Melilite s.s. indicates that under our experimental conditions, the zeta potential remains negative throughout the pH range (from pH 2 to pH 11), and never reaches its point of zero charge (PZC). This result is also similar to the measurement of synthetic gehlenite measured by Udaeta et al.^70^ (for further details on Zeta potential measurement see Supplementary Information, page S18). From the point of view of surface potential, sodium oleate may not be electrostatically adsorbed on Melilite s.s.. Meanwhile, new peaks for oleate adsorption were not observed during IR measurements (see Supplementary Information, page S9).

## Conclusion

In summary it was possible to show that LiAlO_2_ and Melilite s.s. are separable in a froth flotation. Both standard collectors and compounds not previously used as collectors were investigated. Very good yields and selectivities could be achieved with these collectors after optimization. Furthermore, the principle of self-organized monolayers (SAM) was introduced for the first time in a froth flotation system. The pre-functionalization resulted in a significant improvement compared to the standard. These results will allow a new approach for the separation of different minerals via froth flotation in the future and further studies are currently conducted.

## Supplementary Information

Supplementary Information.
