# Supplementary material for: Improvement of the froth flotation of LiAlO2 and melilite solid solution via pre-functionalization
Source: Sci Rep. 2021 Oct 14;11:20443. doi: 10.1038/s41598-021-00008-z (PMC8516992; doi:10.1038/s41598-021-00008-z)
Supplement: Supplementary file 1 — Supplementary Information. [file 41598_2021_8_MOESM1_ESM.pdf]

Supplementary Information

Improvement of the Froth Flotation of $LiAlO_2$ and Melilite solid solution via Pre-Functionalization

Hao Qiu,[1] Jule Kersebaum,[1] Annett Wollmann,[2] Niklas Feuge,[3] Andrea Haas,[1] Daniel Goldmann,[1] & René Wilhelm[3]

[1]Clausthal University of Technology, Institute of Mineral and Waste Processing, Waste Disposal and Geotechnics, Walther-Nernst-Str. 9, 38678 Clausthal-Zellerfeld, Germany. [2]Clausthal University of Technology, Institute of Particle Technology, Leibnizstr. 19, 38678 Clausthal-Zellerfeld, Germany. [3]Clausthal University of Technology, Institute of Organic Chemistry, Leibnizstr. 6, 38678 Clausthal-Zellerfeld, Germany. Correspondence and requests for materials should be addressed to R.W. (email: rene.wilhelm@tu-clausthal.de) or D.W. (email: daniel.goldmann@tu-clausthal.de)

Froth Flotation Parameter Optimization for Sodium Oleate and Sodium Naphthenate

Effect of Collector Dosage on Flotation of $LiAlO_2$ and Melilite s.s. with Sodium Oleate. Figure S1 presents the effects of collector dosage on the yield of $LiAlO_2$ in the Hallimond tube flotation with sodium oleate at ambient temperature and at natural pH, pH 7, and pH 9. The natural pH of $LiAlO_2$ is about 11. As shown in Figure S1, the yield increased gradually with the increase of collector dosage from 50 g/t to 200 g/t. A slight decrease in yield was then observed, when the dosage was increased from 200 to 250 g/t. The best result was achieved with a collector dosage of 300 g/t at natural pH with a yield of 69.55%. In most cases, the flotation results at natural pH are higher than those at pH 7 and pH 9. On the other hand, no significant increase in yield with increasing collector dosage was observed at pH 7 and pH 9. Nevertheless, the results at pH 9 are slightly higher than the results at pH 7.

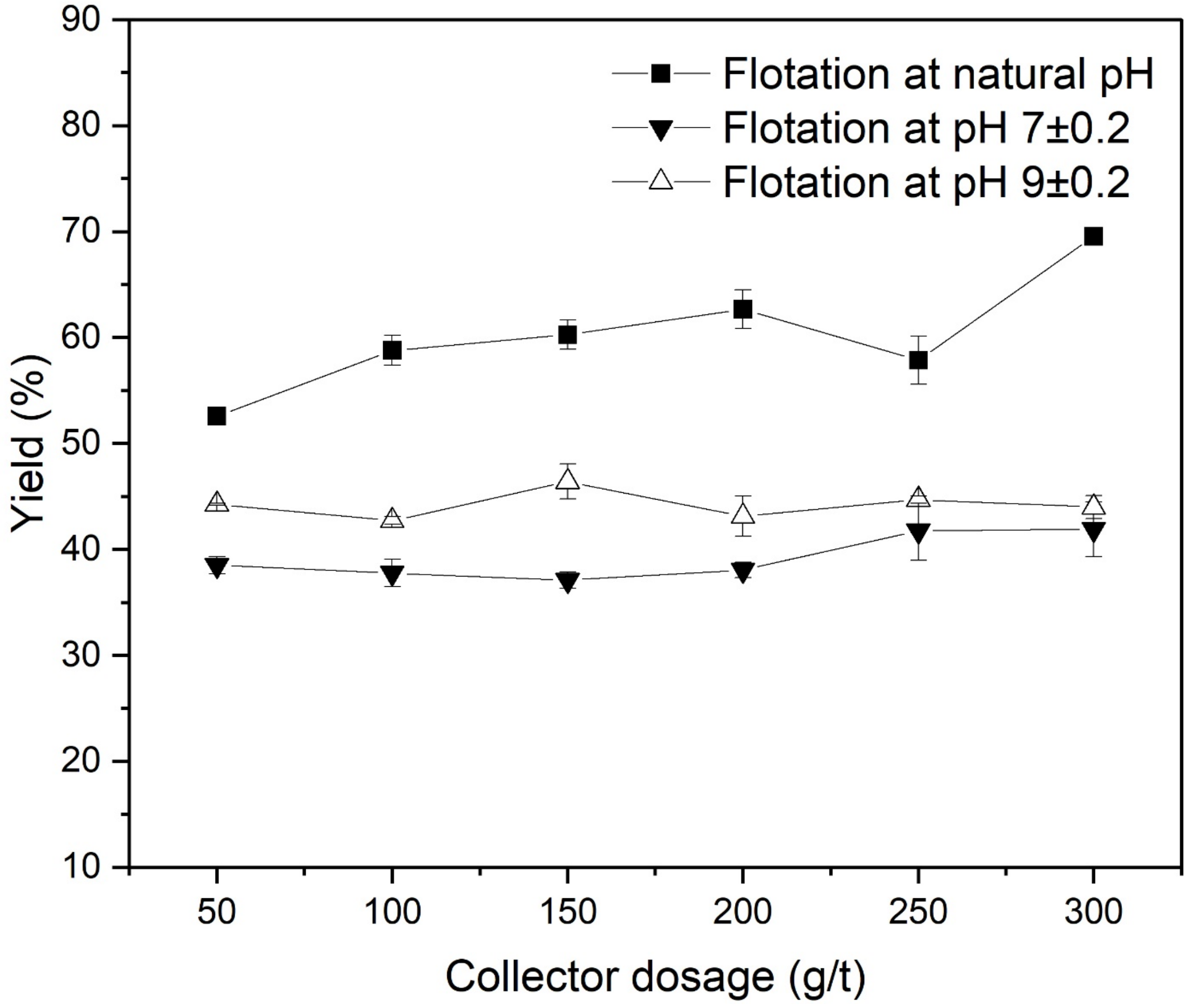

Figure S1. Effect of Collector Dosage on Flotation of $LiAlO_2$ using Sodium Oleate

Figure S2 presents the effects of collector dosage on the yield of Melilite s.s. in the Hallimond tube flotation with sodium oleate at ambient temperature and at natural pH, pH 7 and pH 9. The natural pH of Melilite s.s. is about 10. As shown in Figure S1, when the dosage of sodium oleate was 300g/t, the yields at all three pH were lower than those of $LiAlO_2$. The highest yield was achieved with a collector dosage of 1000 g/t at pH 7 with a yield of 48.01%. In the combination of Figures. S1 and S2, it may be seen that sodium oleate showed relatively good selectivity for the flotation of $LiAlO_2$. The yield of Melilite s.s. is about 34% lower than that of $LiAlO_2$.

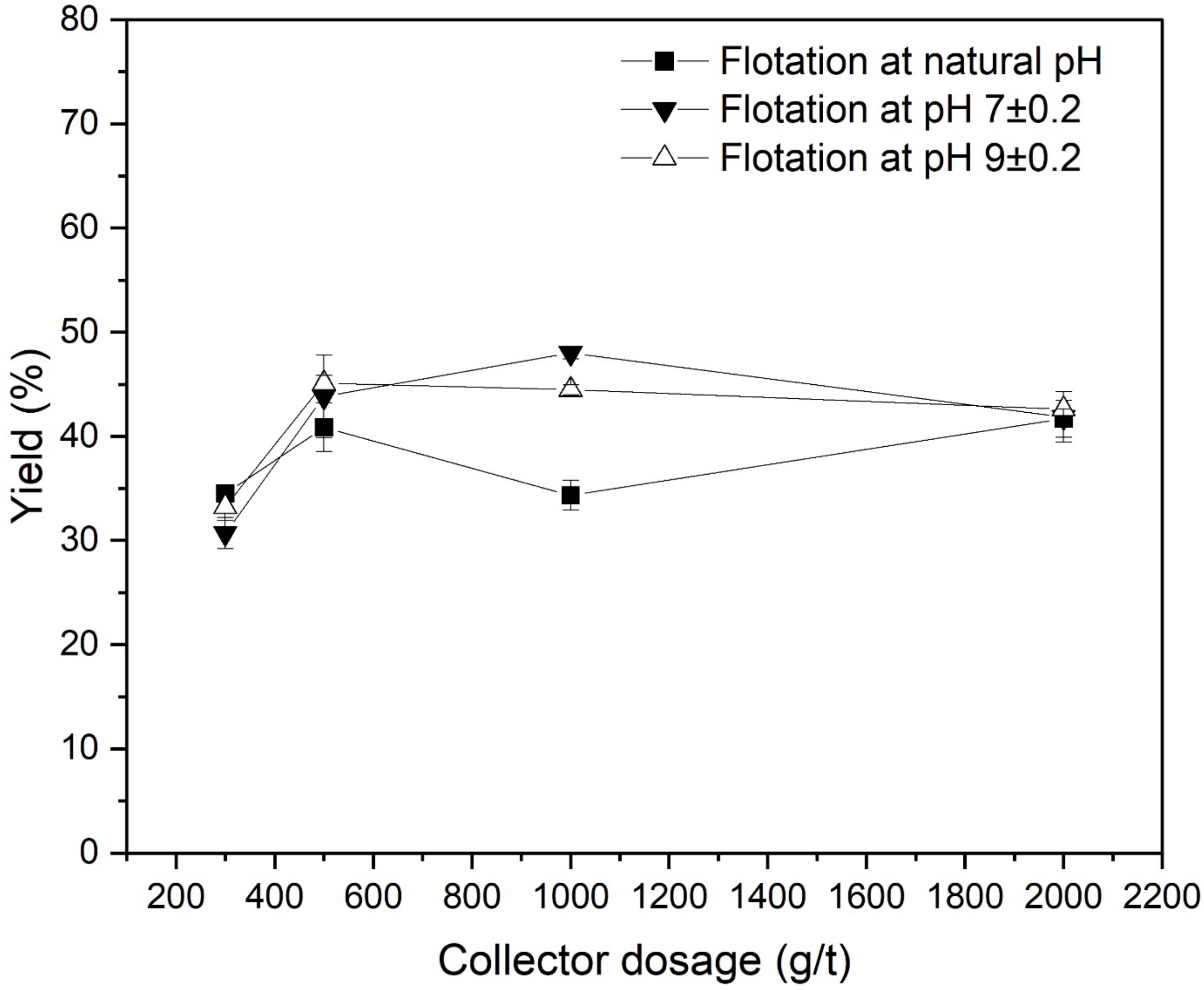

Figure S2. Effect of Collector Dosage on Flotation of Melilite s.s. using sodium oleate

Simultaneously, the yields of $LiAlO_2$ at natural pH were higher than those at pH 7 and pH 9, which may be attributable to the solution equilibriums of oleic acid. The species distribution diagram of oleic acid at a concentration of $6 \cdot 10^{-4}$ M is shown in Figure S3[2,3] and helps explain the results. According to the diagram, concentrations of oleic ion monomer and dimer remain constant above pH 7.45 and then decrease sharply below 7.45. The concentrations of oleic

molecules (RCOOH) and oleic ionic-molecular ($RCOOH \cdot RCOO^{-}$) decrease sharply when the pH was further increased from 7 to 12. Since the natural pH of $LiAlO_2$ is around 11, a massive amount of oleic ion monomers and dimers should be present in the solution. Combined with the IR-spectra analysis, it may be due to the chemisorption of oleic ion monomer and dimer with aluminum ions on the surface of $LiAlO_2$, which causes the yield of $LiAlO_2$ at natural pH higher than those at pH 7 and pH 9.

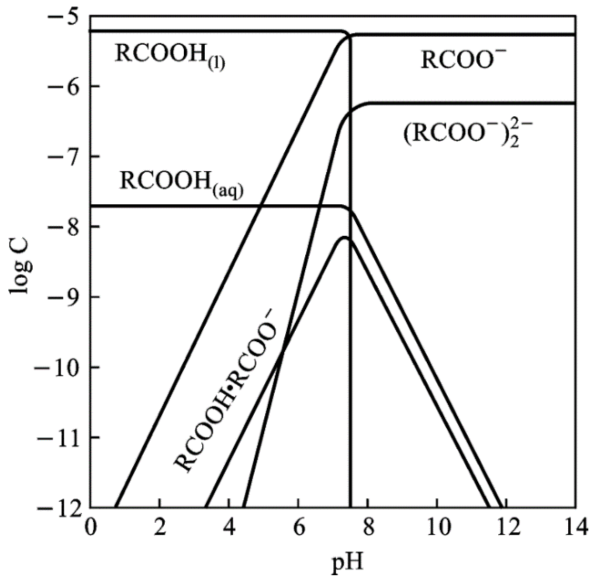

Figure S3. Species distribution diagram of oleic acid as a function of pH for 6*10-4 M aqueous solution.[2,3]

Effect of Collector Dosage on Flotation of $LiAlO_2$ and Melilite s.s. with sodium naphthenate Figure S4 presents the effects of collector dosage on the yield of $LiAlO_2$ in the Hallimond tube flotation with sodium naphthenate at ambient temperature and at natural pH, pH 7, and pH 9. As shown in Figure S4, the best result was achieved with a collector dosage of 250 g/t at natural pH with a yield of 60.17%. The best result is slightly lower than that of sodium oleate, and the flotation results at natural pH are overall higher than the results at pH 7 and pH 9. The yield increased gradually with the increase of collector dosage from 50 g/t to 250 g/t. However, if the dosage continued to be increased from 250 to 300 g/t, a slight decrease of yield was observed. In most cases, a significant increase of yield was not observed while increasing the dosage of the collector at pH 9. Nevertheless, the results at pH 9 are slightly higher than the results at pH 7. The yield increased steadily with the rise of dosage at pH 7.

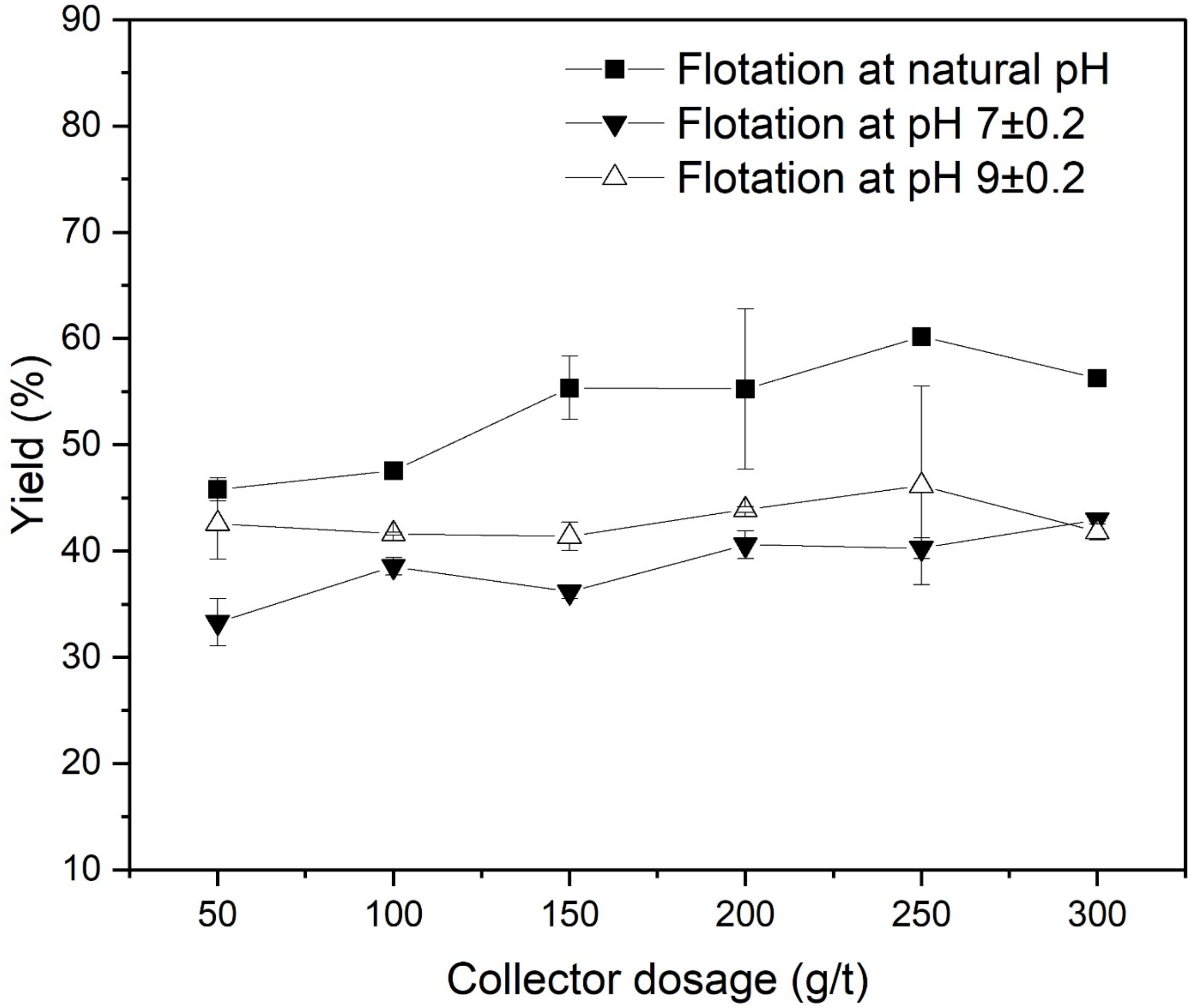

Figure S4. Effect of Collector Dosage on Flotation of $LiAlO_2$ using sodium naphthenate

Figure S5 presents the effects of collector dosage on Melilite s.s. yield in the Hallimond tube flotation with sodium naphthenate at ambient temperature and at natural pH, pH 7 and pH 9. As shown in Figure S5, when the dosage of Sodium naphthenate was 300g/t, the yields at all three pHs were lower than those of $LiAlO_2$. The highest yield was achieved with a collector dosage of 2000 g/t at natural pH with a yield of 48.37%. Combining Figures S4 and S5, it can be seen that for the flotation of $LiAlO_2$ sodium naphthenate showed relatively specific selectivity. The flotation selectivity is merely lower than sodium oleate. The yield of Melilite s.s. with a dosage of 300 g/t is about 24% lower than that of $LiAlO_2$.

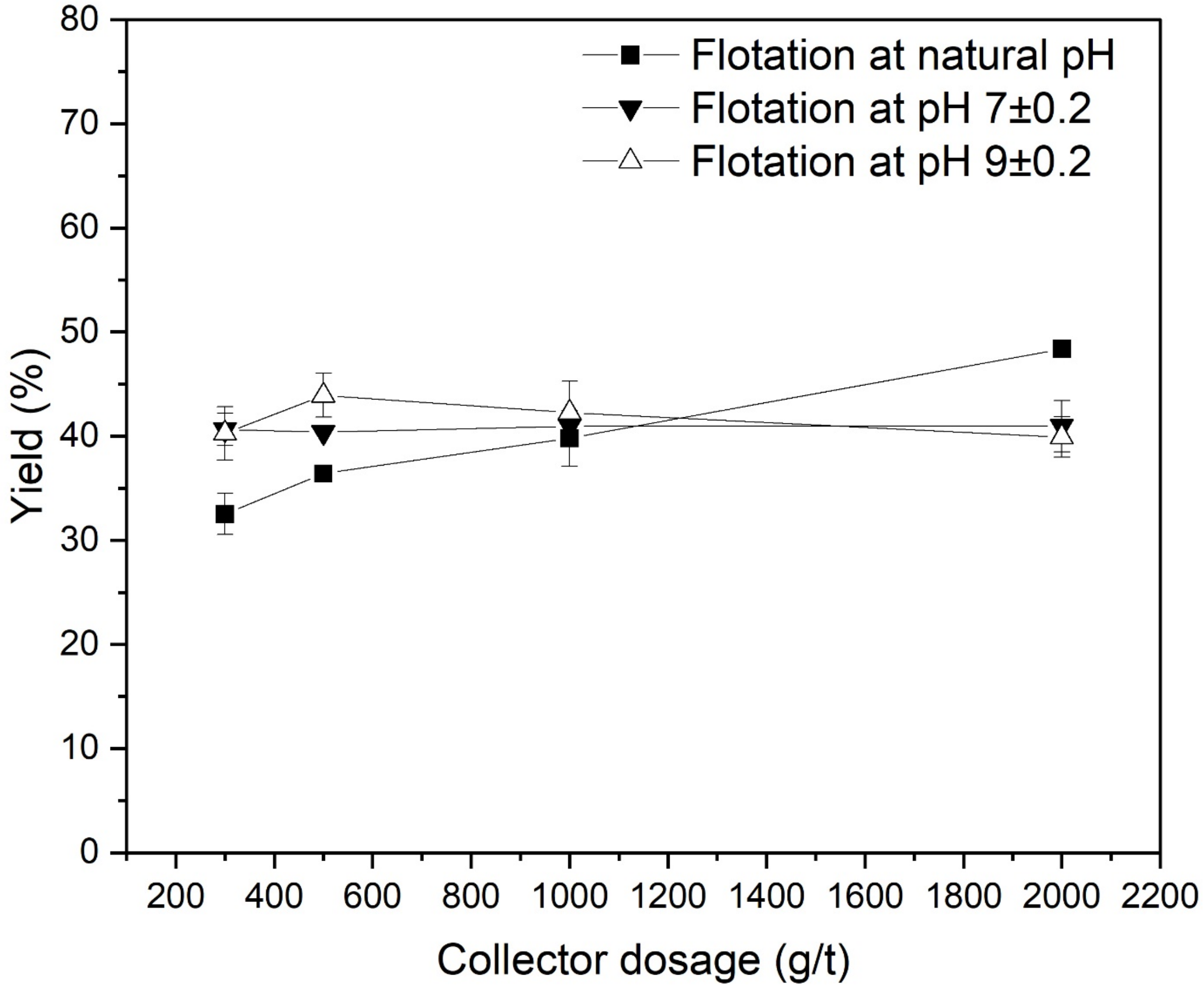

Figure S5. Effect of Collector Dosage on Flotation of Melilite s.s. using sodium naphthenate

TGA measurements and Elemental Analysis results

TGA example of Melilite s.s.

Due to the water content in the silicate, the non-treated Melilite s.s. showed a much higher continuous weight loss due to incorporated water in the silicate. Hence, further studies with Melilite s.s. concentrated on Elemental Analysis

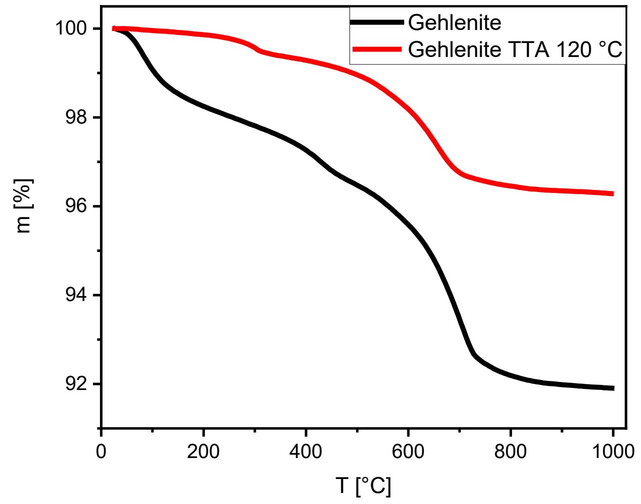

Figure S6. TGA of Melilite s.s. and Melilite functionalized with TTA

TGA measurements $LiAlO_2$ functionalized with TTA and dodecyl phosphonic acid

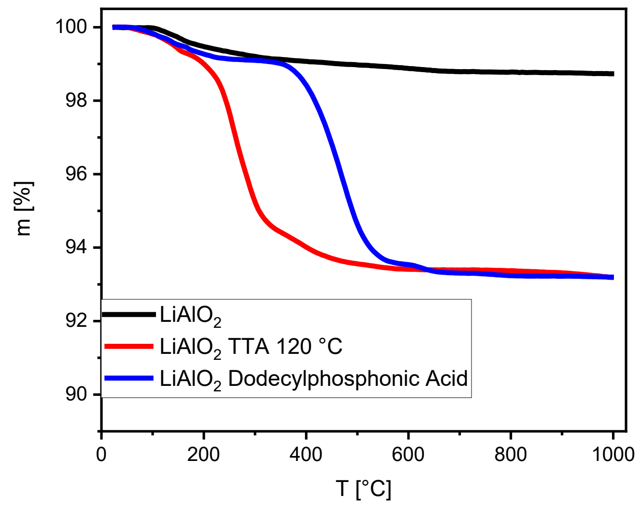

Figure S7. TGA of pure $LiAlO_2$ and functionalized with TTA and dodecyl phosphonic acid

Elemental Analysis

Loading was calculated considering the carbon content from a sample and the molecular mass of the molecule. The results for functionalized $LiAlO_2$ are shown in the table below. Since it is not known in which angle the molecules are aligned on the surface, precise calculations on the surface coverage are not possible. However, the loading is in complete alignment with the observed results in the flotation. Dodecyl phosphonic acid showed even at room temperature functionalization a high hydrophobic behavior. The flotation experiment with TTA was improved compared to sodium oleate, which again gave better results than TOPO.

**Sample**	**Carbon content [%]**	**Loading [mmol/g]**
$LiAlO_2$ + TOPO 120 °C	0.30	0.01
$LiAlO_2$ + dodecylphosphonic acid rt	5.73	0.40
$LiAlO_2$ + TTA 120 °C	2.36	0.25
$LiAlO_2$ + sodium oleate 120 °C	1.66	0.08

Table S1. Elemental Analysis of functionalized $LiAlO_2$

The corresponding results for Melilite s.s. are given in the table below. Taking into consideration that the BET of $LiAlO_2$ was <1 m^2/g and for Melilite s.s. 3.4 m^2/g one would need to divide the loadings below through at least 3.4 to compare these results with $LiAlO_2$. Even without this consideration, one can find a significantly lower loading of TTA on Melilite.

**Sample**	**Carbon content [%]**	**Loading [mmol/g]**
Melilite s.s. + TOPO 120 °C	0.11	0.004
Melilite s.s. + TTA 120 °C	0.71	0.07
Melilite s.s. + sodium oleate r.t.	1.04	0.05

Table S2. Elemental Analysis of functionalized Melilite s.s.

Further FT-IR spectra

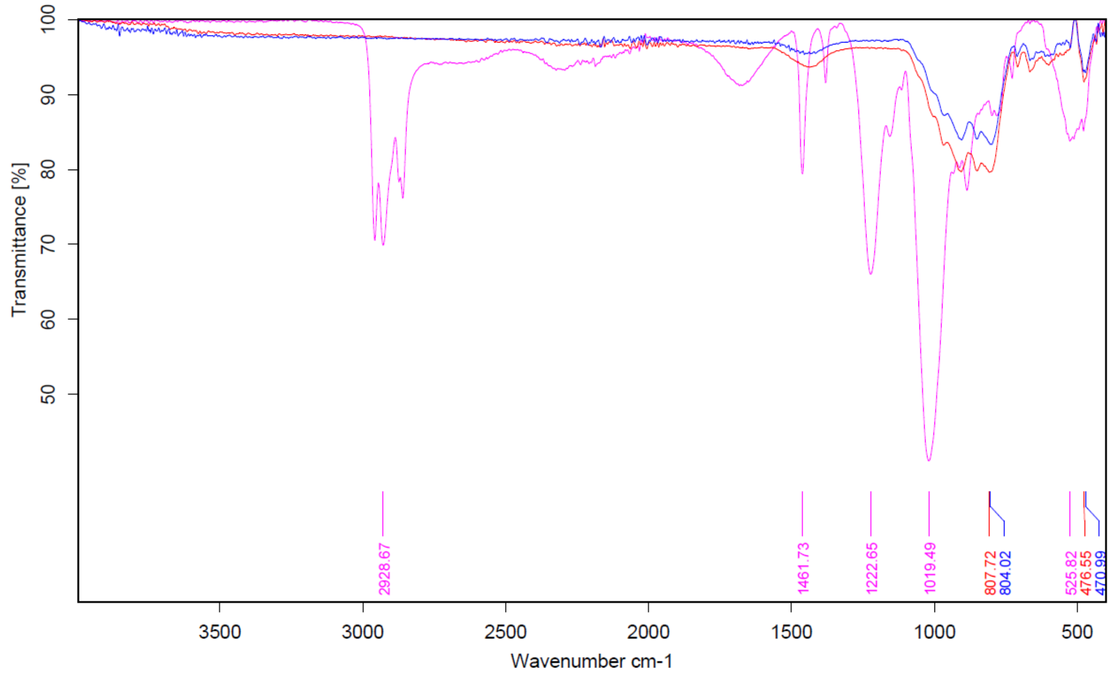

Sample Name Gehl_Bis	Sample Form Instrument type and / or accessory
Sample Name MS_Gehl_rein	Sample Form Instrument type and / or accessory
Sample Name Bis-2-ethyl-hexylphosphat	Sample Form Instrument type and / or accessory

Figure S8. Bis-2-ethyl-hexyl phosphate pure, Melilite s.s. pure, material from standard flotation

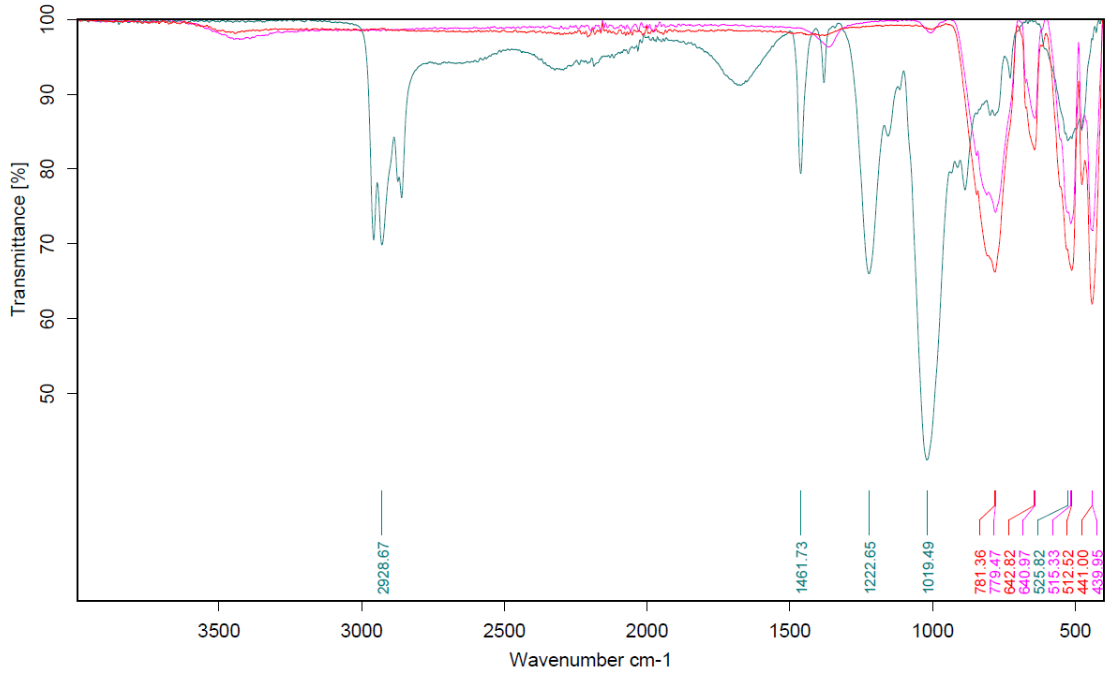

Sample Name MS_Li_rein	Sample Form Instrument type and / or accessory
Sample Name Bis-2-ethyl-hexylphosphat	Sample Form Instrument type and / or accessory
Sample Name Li_Bis	Sample Form Instrument type and / or accessory

Figure S9. Bis-2-ethyl-hexyl phosphate pure, $LiAlO_2$ pure, material from standard flotation

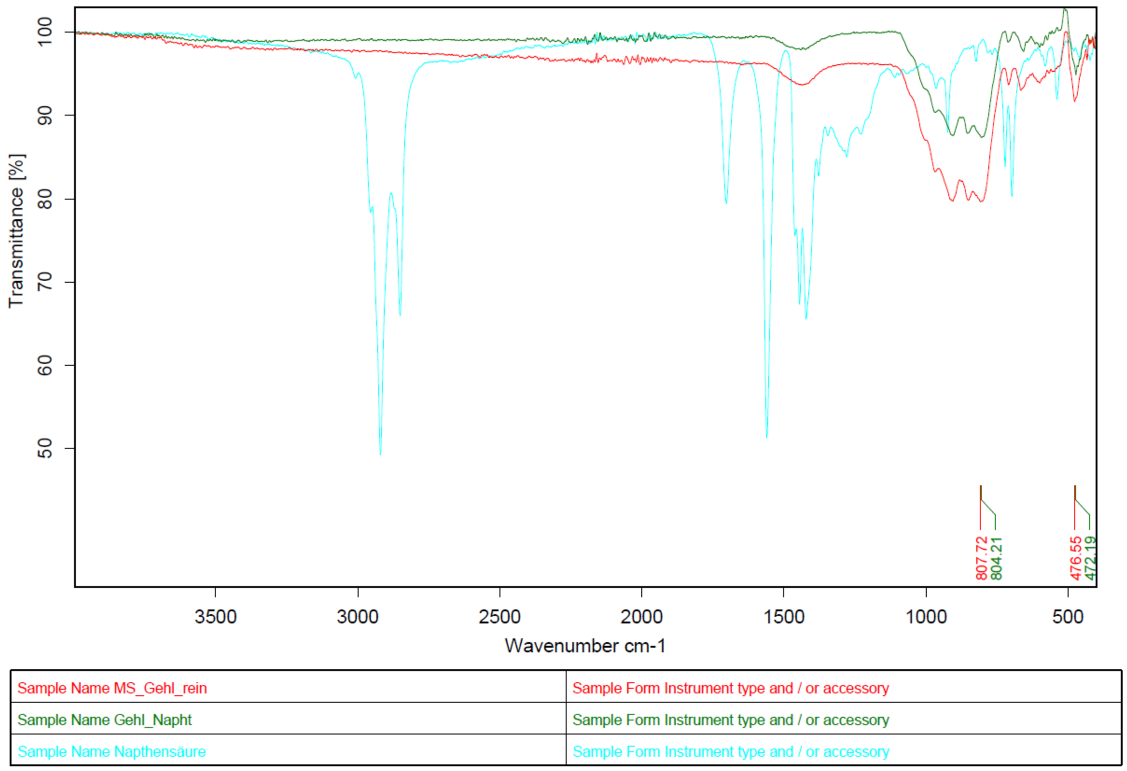

Figure S10. Sodium naphthenoate pure, Melilite s.s. pure, material from standard flotation

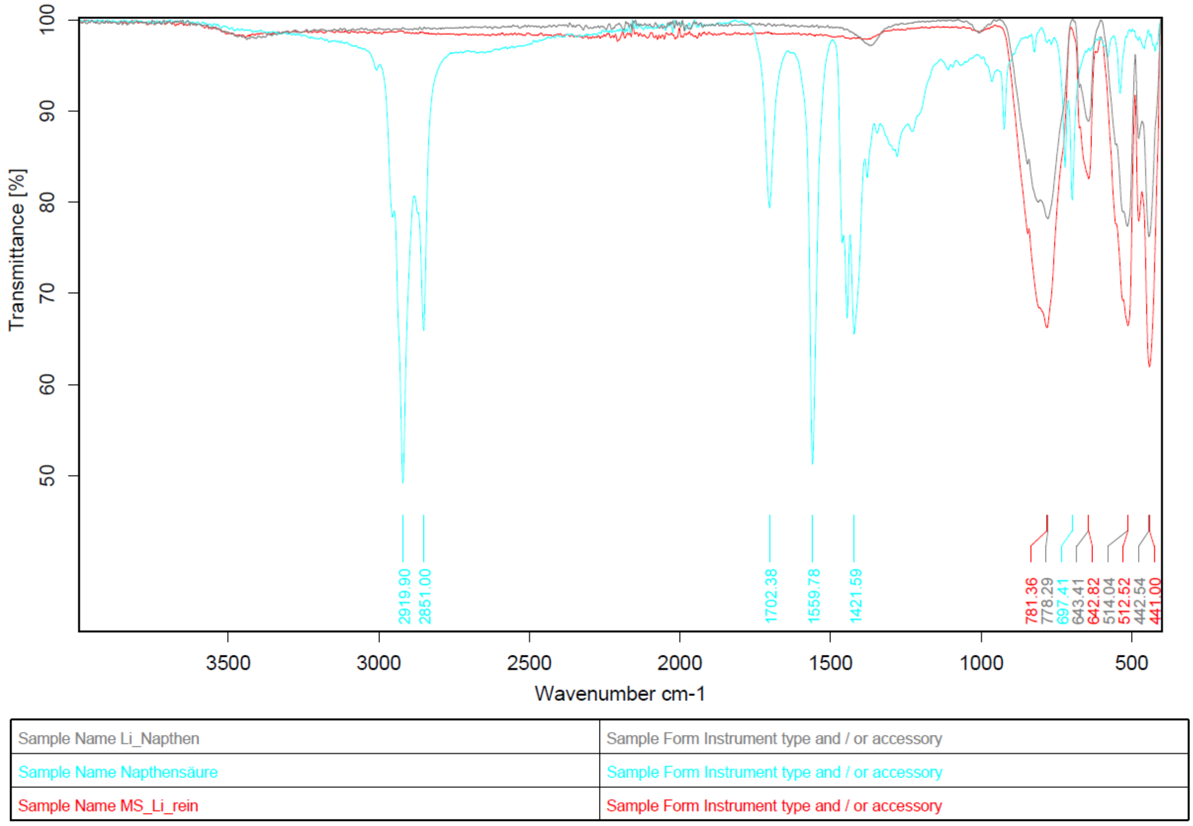

Figure S11. Sodium naphthenoate pure, $LiAlO_2$ pure, material from standard flotation

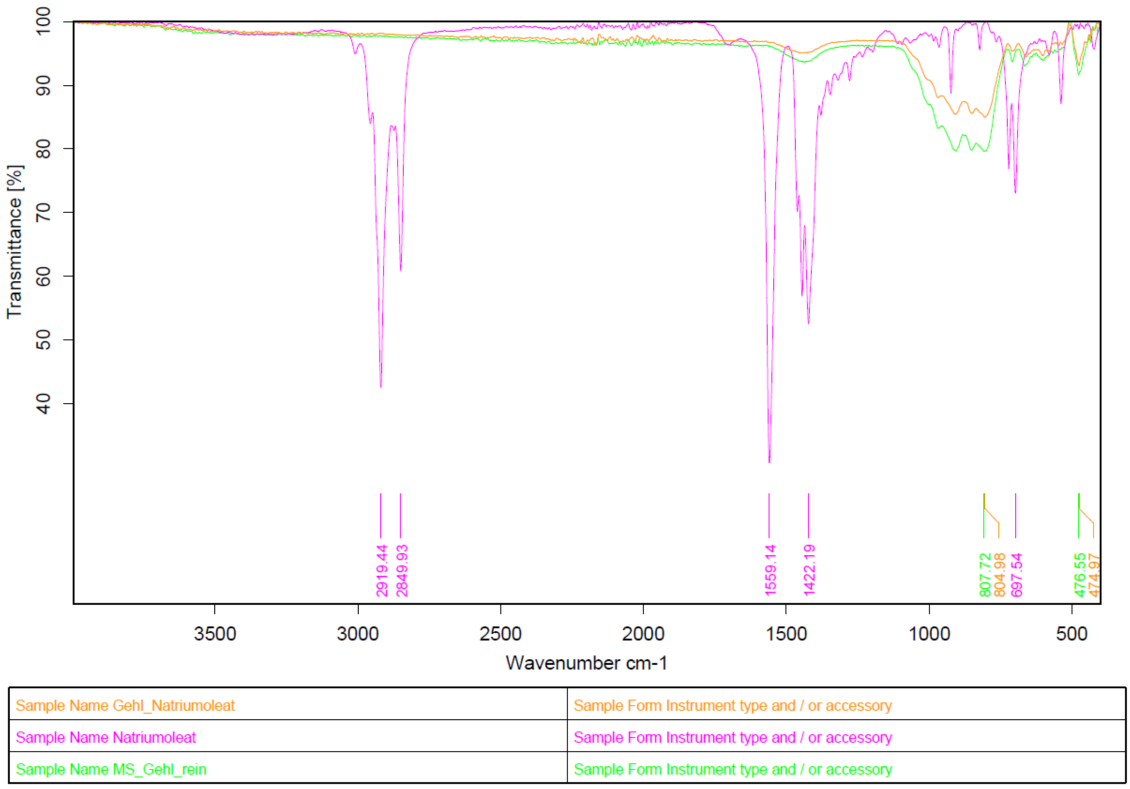

Figure S12. Sodium oleate pure, Melilite s.s. pure, material from standard flotation

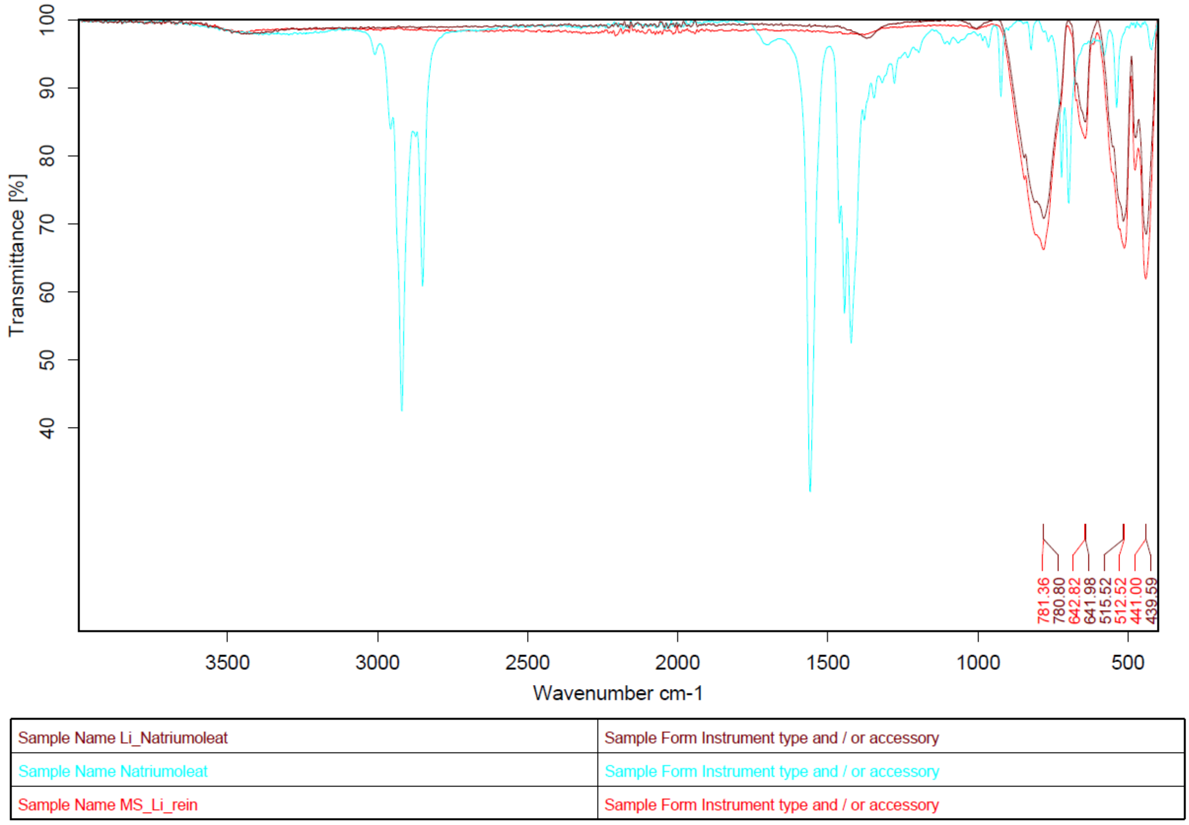

Figure S13. Sodium oleate pure, $LiAlO_2$ pure, material from standard flotation

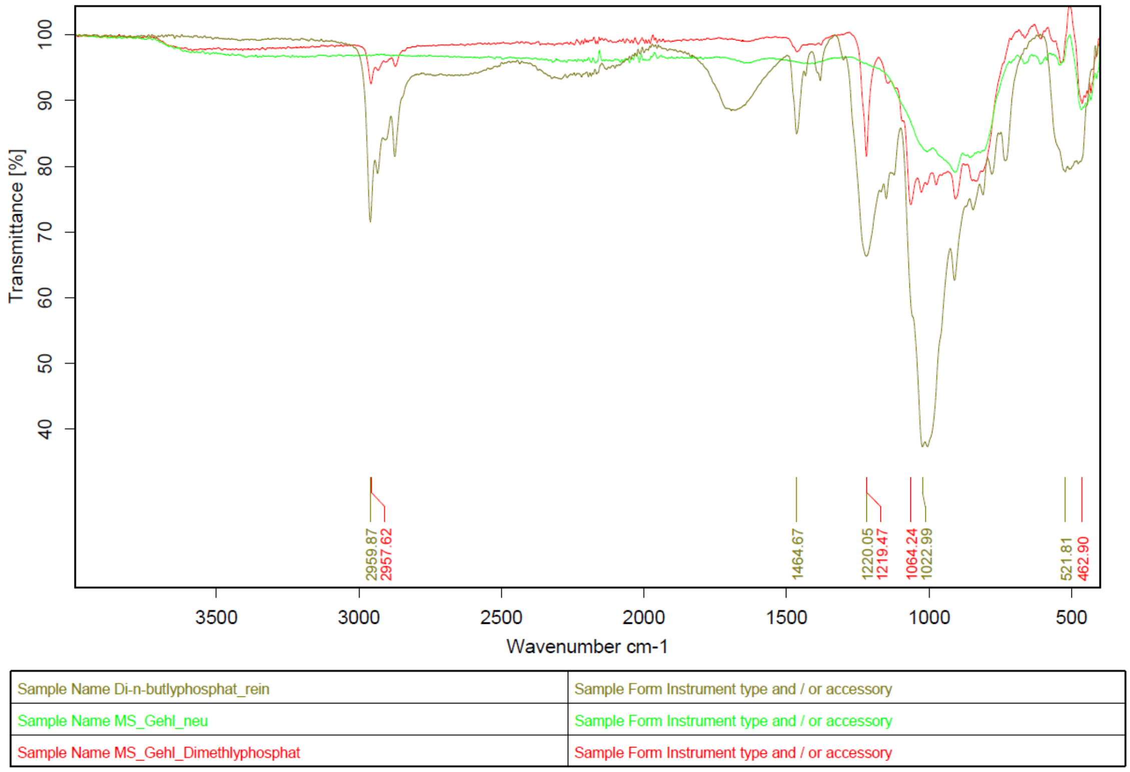

Figure S14. Di-n-butyl phosphate pure, Melilite s.s. pure, functionalized at 120 °C

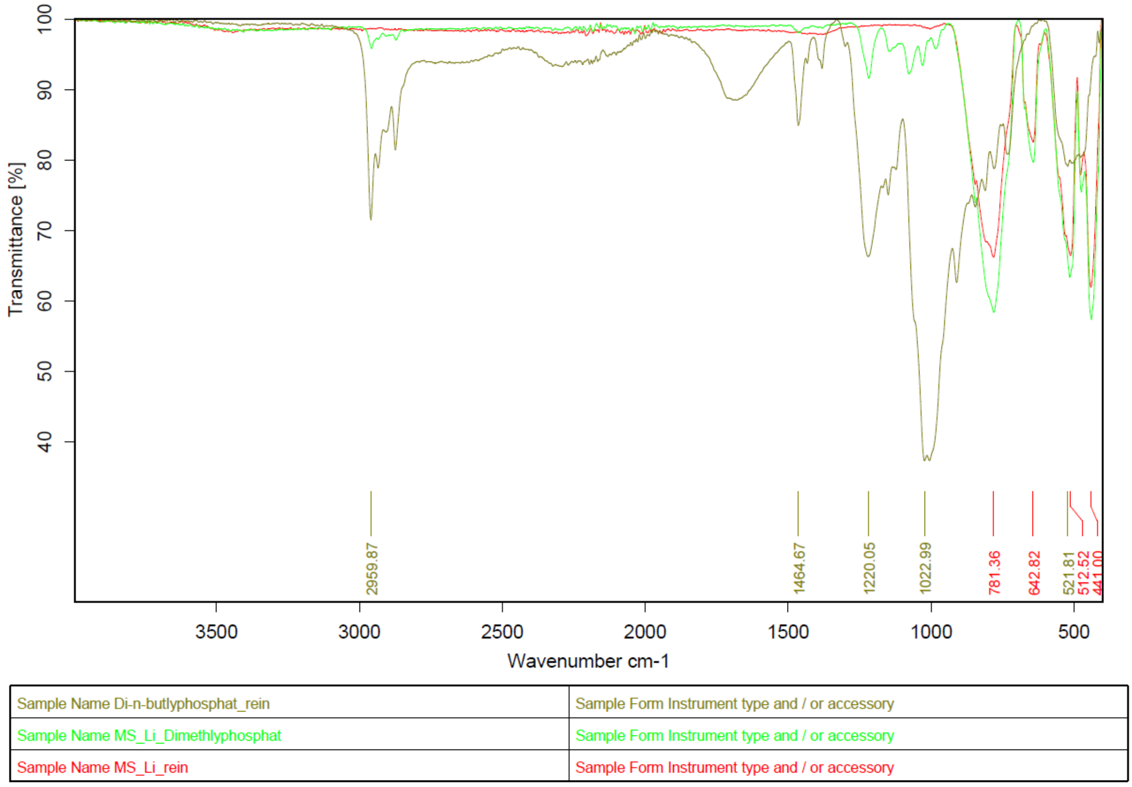

Figure S15. Di-n-butyl phosphate pure, $LiAlO_2$ pure, functionalized at 120 °C

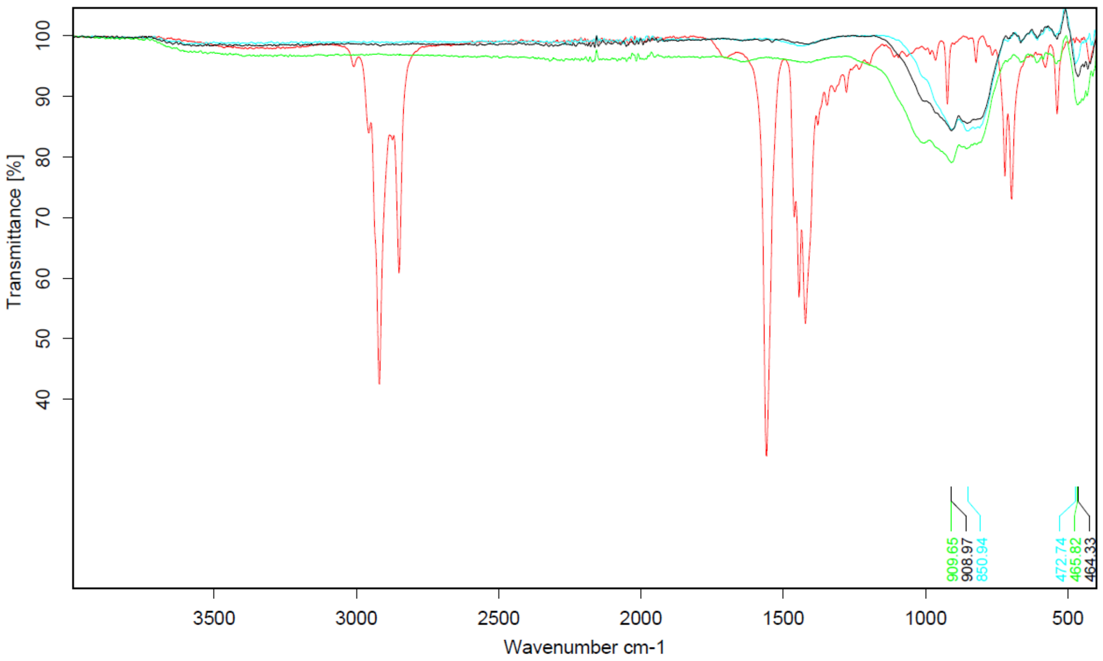

Sample Name Natriumoleat_Gehl_RT	Sample Form Instrument type and / or accessory
Sample Name MS_Gehl_neu	Sample Form Instrument type and / or accessory
Sample Name Natriumoleat_Gehl_120	Sample Form Instrument type and / or accessory
Sample Name Natriumoleat	Sample Form Instrument type and / or accessory

Figure S16. Sodium oleate pure, Melilite s.s. pure, functionalized at r.t. and 120°C

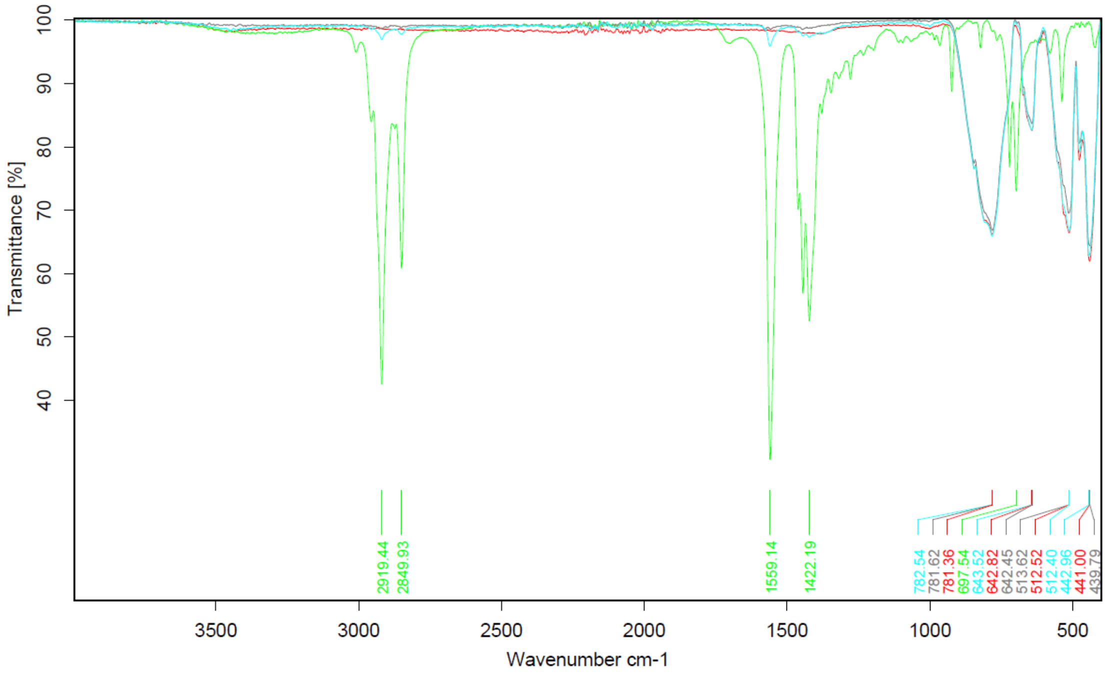

Sample Name Natriumoleat_Li_RT	Sample Form Instrument type and / or accessory
Sample Name Natriumoleat_Li_120	Sample Form Instrument type and / or accessory
Sample Name Natriumoleat	Sample Form Instrument type and / or accessory
Sample Name MS_Li_rein	Sample Form Instrument type and / or accessory

Figure S17. Sodium oleate pure, $LiAlO_2$ pure, functionalized at r.t. and 120°C

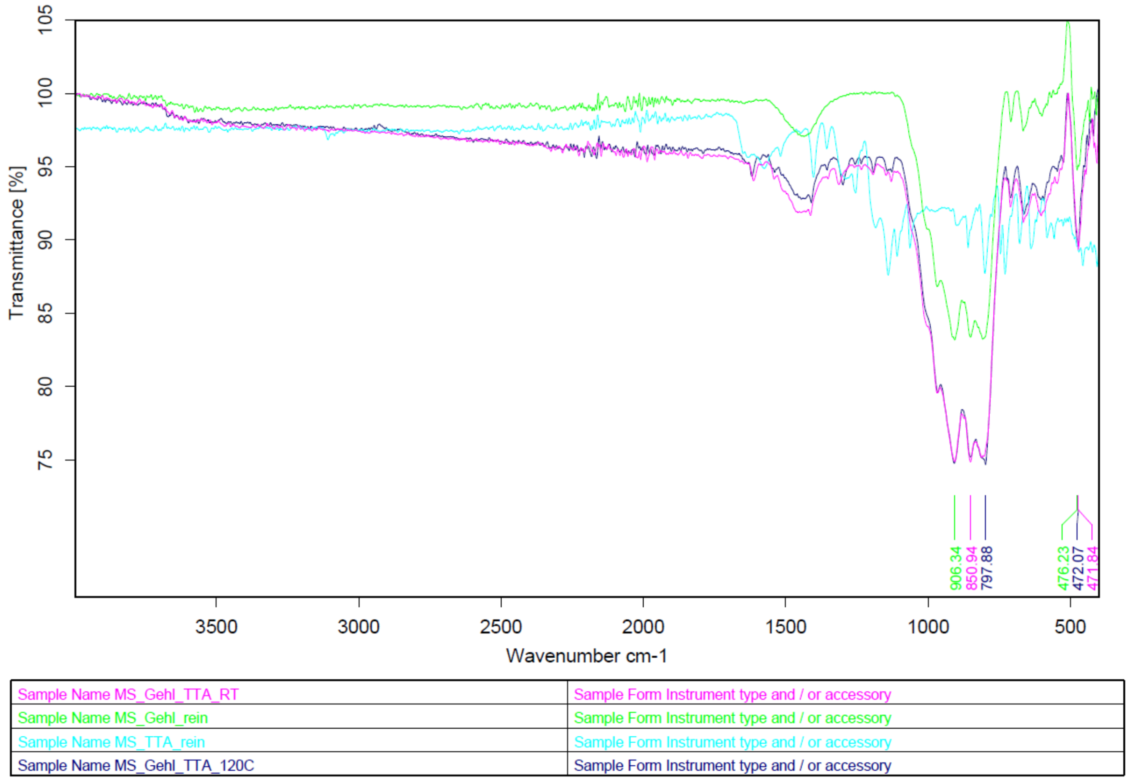

Figure S18. TTA pure, Melilite s.s. pure, functionalized at r.t. and 120°C

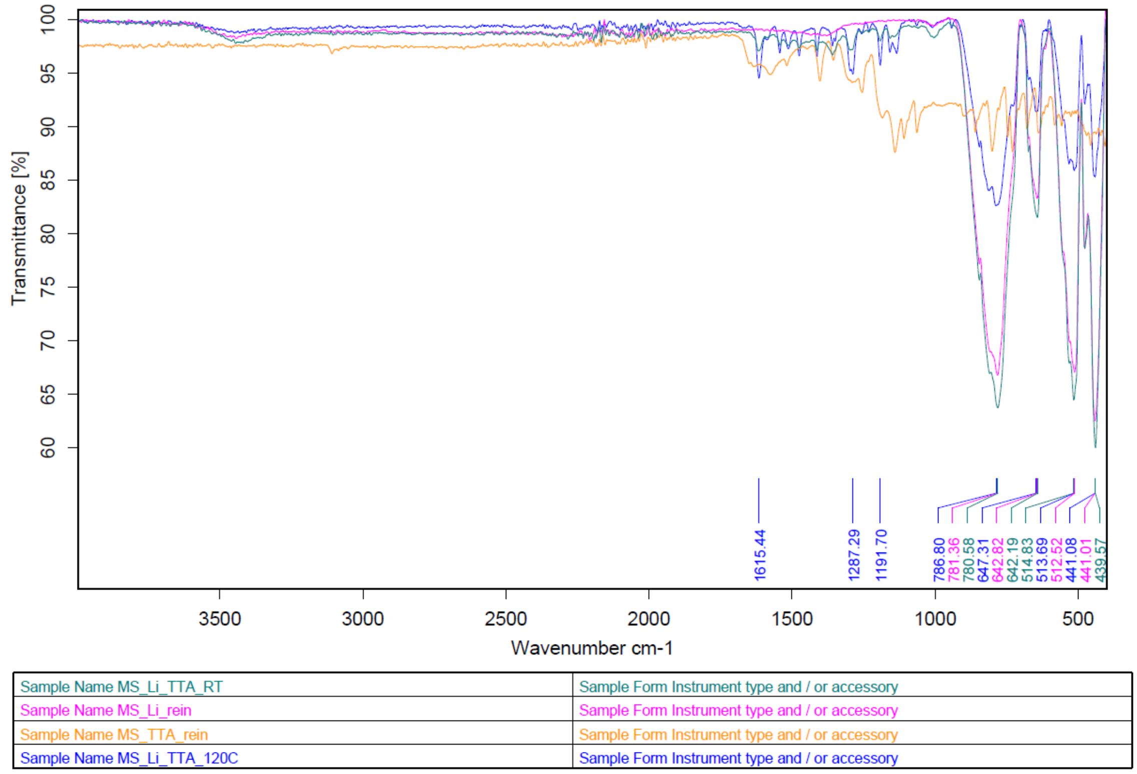

Figure S19. TTA pure, $LiAlO_2$ pure, functionalized at r.t. and 120°C

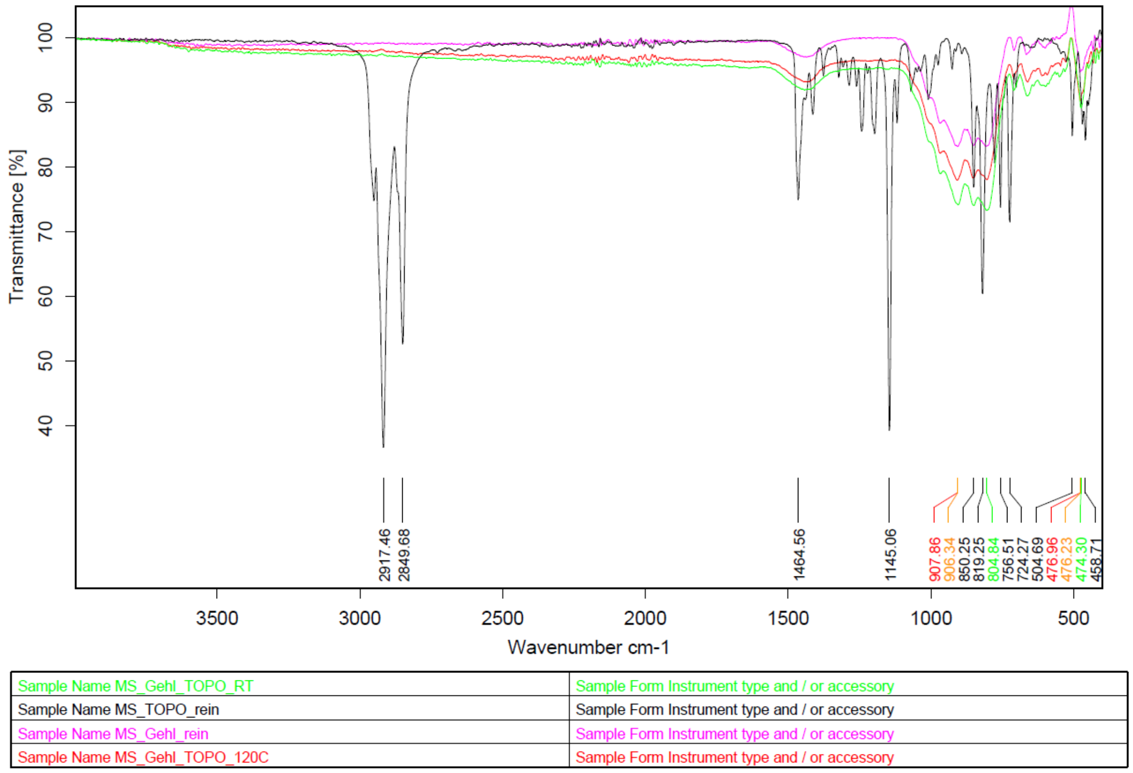

Figure S20. TOPO pure, Melilite s.s. pure, functionalized at r.t and 120 °C

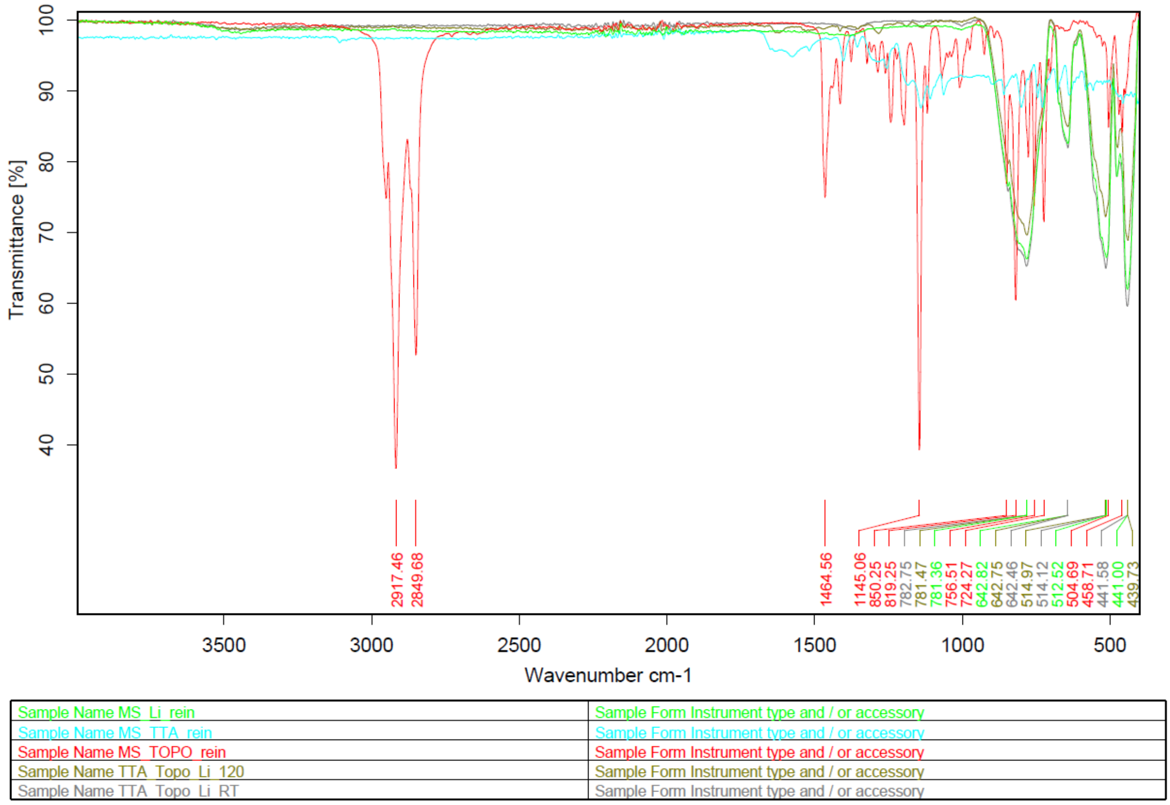

Figure S21. TTA pure, TOPO pure, $LiAlO_2$ pure, functionalized at r.t and 120 °C

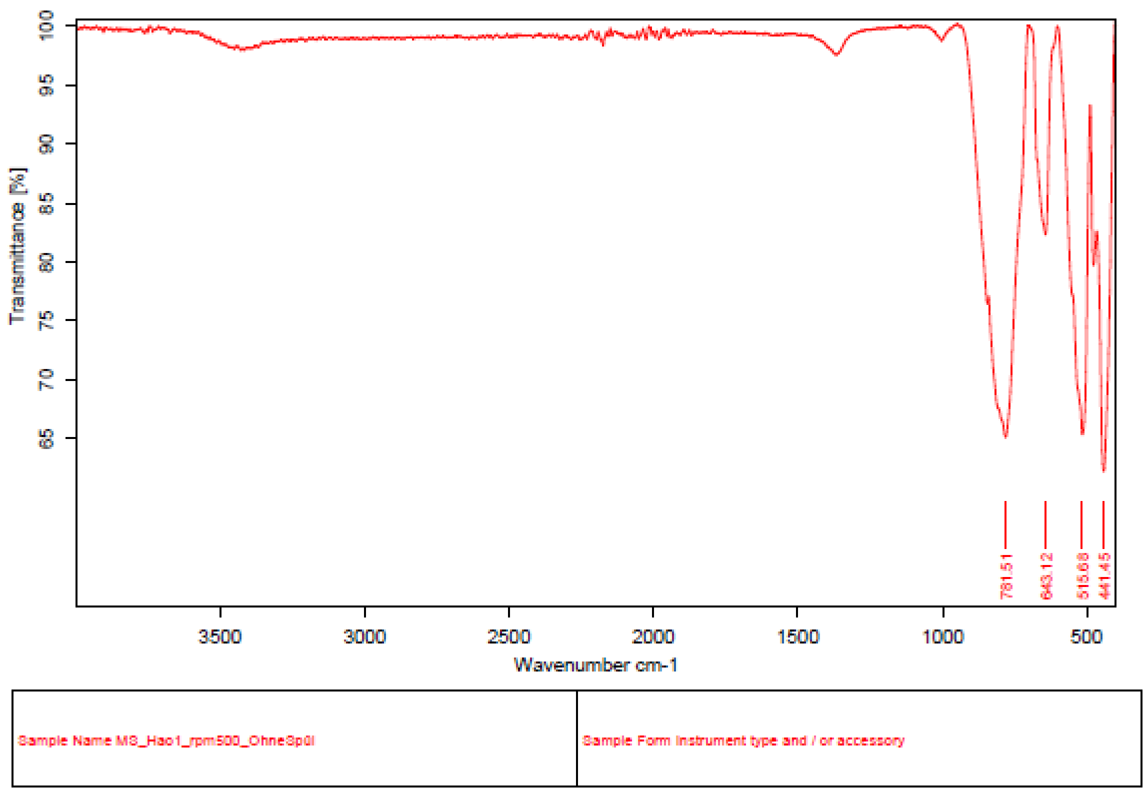

Figure S22. rpm 500, sodium oleate

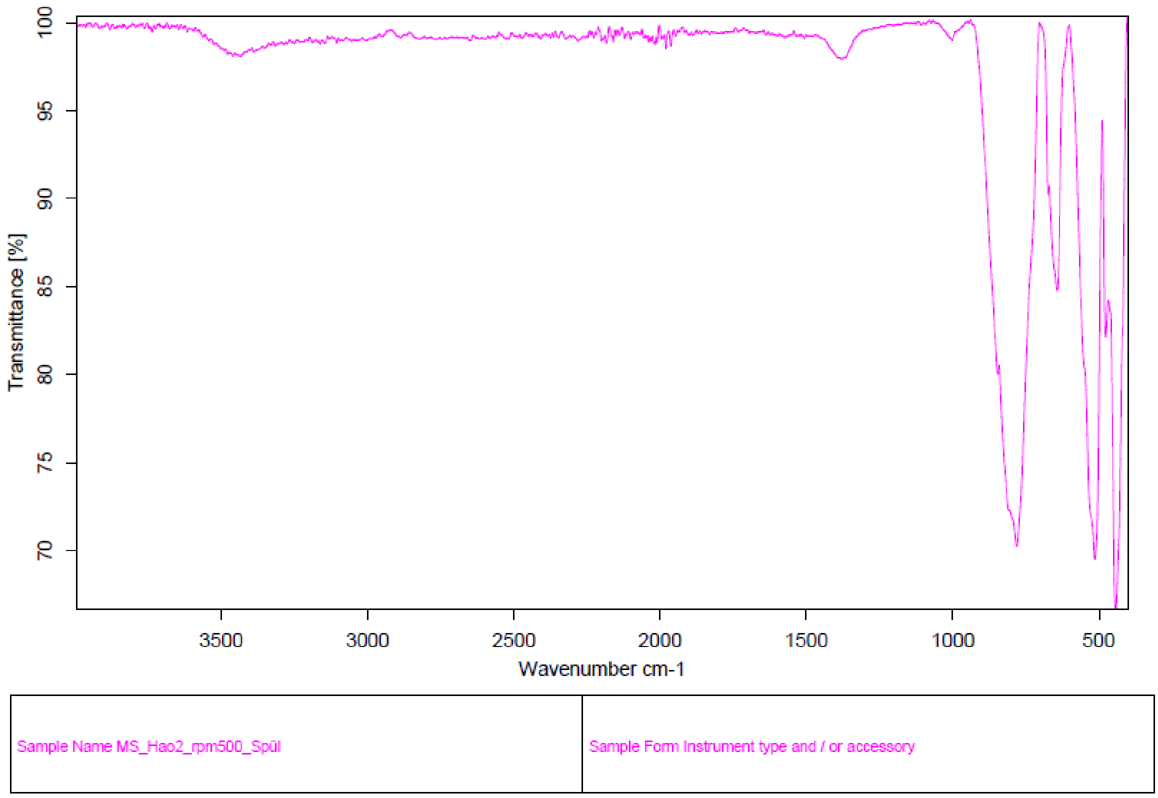

Figure S23. rpm 500, sodium oleate, washed with distilled water

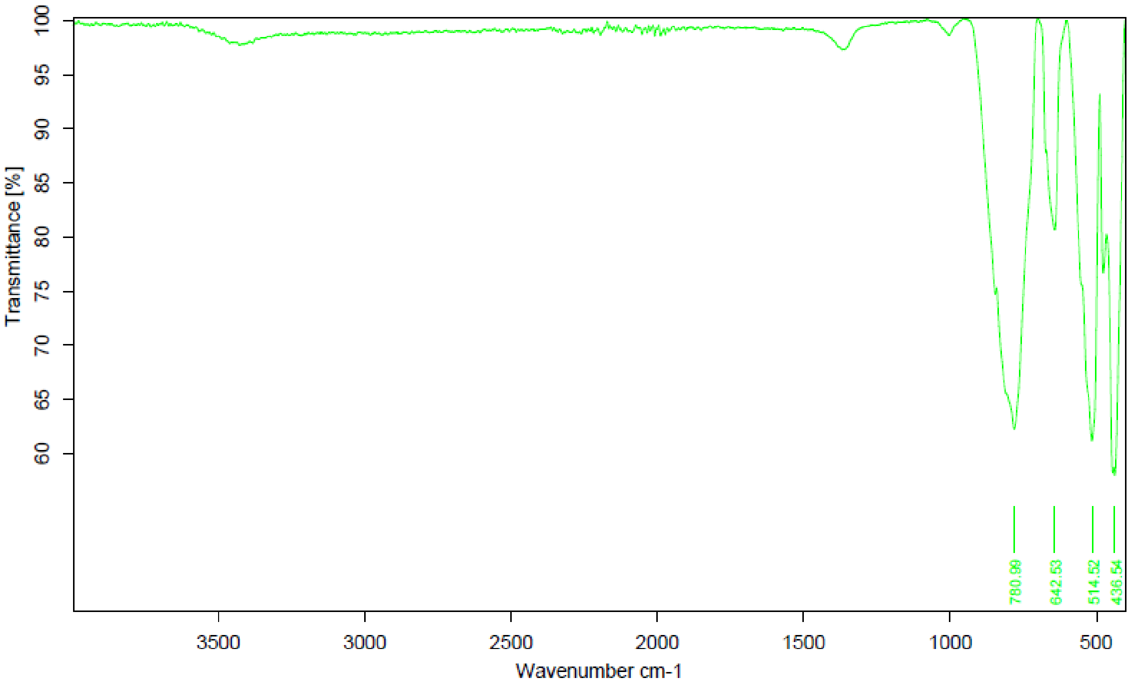

Sample Name MS_Hao3_rpm1000_Spül	Sample Form Instrument type and / or accessory

Figure S24. Sodium oleate, rpm 1000, washed with distilled water

Flotation Results of Material functionalized with sodium oleate and oleic acid

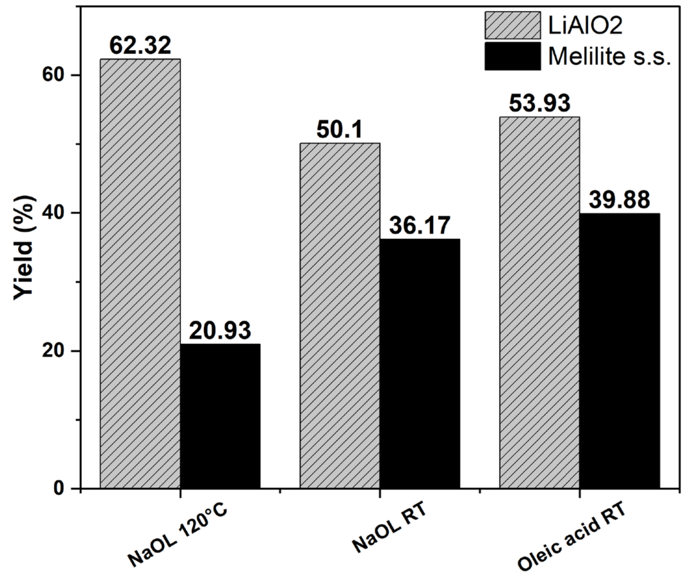

Figure S25. Flotation yield of NaOL and oleic acid functionalized $LiAlO_2$ and Melilite s.s.

Zeta Potential Measurement

Zeta potential was measured in a Zetasizer Nano (Malvern) with an automatic titrator mpt-2. A 1 mM KCl solution was chosen as the electrolyte. A 0.333 g Melilite s.s. sample was placed in a 500 mL measuring cylinder and electrolyte was added to 500 mL. The pulp was allowed to stand for 39 minutes after being agitated. The supernatant was taken for zeta potential measurement. During the measurement, the pH was adjusted with 1 M HCl and 1 M NaOH.

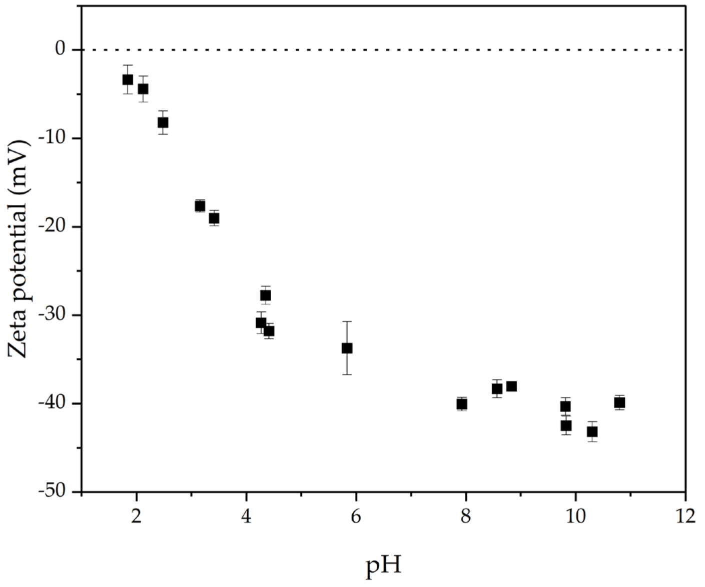

Figure S26. Zeta potential of Melilite s.s.

References

1 Okamoto, Y. Synthesis of Alkyl Dihydrogenphosphate by the Reaction of Alcohols and Silyl Polyphosphate. *Bull. Chem. Soc. Jpn.* **58**, 3393-3394 (1985).

2 Somasundaran, P. & Wang, D. *Solution chemistry. Minerals and reagents*. 1 edn, Vol. 17 (Elsevier Science, 2006).

3 Somasundaran, P., Ananthapadmanabhan, K. P. & Ivanov, I. B. Dimerization of oleate in aqueous solutions. *J. Colloid Interface Sci.* **99**, 128-135 (1984).
